# Spatial Neglect Subtypes, Definitions and Assessment Tools: A Scoping Review

**DOI:** 10.3389/fneur.2021.742365

**Published:** 2021-11-24

**Authors:** Lindy J. Williams, Jocelyn Kernot, Susan L. Hillier, Tobias Loetscher

**Affiliations:** ^1^Cognitive Aging and Impairment Neurosciences Lab, University of South Australia, Adelaide, SA, Australia; ^2^Innovation IMPlementation and Clinical Translation (IIMPACT) in Health, University of South Australia, Adelaide, SA, Australia; ^3^Allied Health and Human Performance, University of South Australia, Adelaide, SA, Australia; ^4^Justice and Society, University of South Australia, Adelaide, SA, Australia

**Keywords:** perceptual disorders, stroke, patient outcome assessment, neuropsychological test, spatial neglect, neglect

## Abstract

**Objective:** The objective of this scoping review was to capture the reported definitions for the subtypes of neglect post stroke and map the range of assessment tools employed for each neglect subtype.

**Methods:** EMBASE, Emcare, Medline, and psychINFO were searched from database inception. Searching included all allied terms and mesh headings for stroke, spatial neglect, measurement, screening tools, psychometric properties. Two reviewers independently screened studies for inclusion. Primary studies with documented protocols of a spatial neglect tool for adults post stroke, with some aspect of validity or reliability were included. Two reviewers independently reviewed the documented protocols of each tool to determine the underlying subtypes and disagreements were resolved through discussion.

**Results:** There were 371 articles included with 292 tools used for the screening or diagnosis of neglect. The majority of studies (67%) included a tool that did not specify the neglect subtype being assessed, therefore an analysis of the underlying subtypes for each tool is presented.

**Conclusions:** There is no consistency with the terms used to refer to the syndrome of spatial neglect with over 200 different terms used within the included studies to refer to the syndrome as a whole or one of its subtypes. It is essential to unify the terminology and definition for each neglect subtype. There are hundreds of neglect tools available, however many are not able to differentiate presenting subtypes. It is important for clinicians and researchers to critically evaluate the neglect tools being used for the screening and diagnosis of neglect.

## Introduction

One in four adults in their lifetime will have a stroke (1). Stroke commonly results in spatial neglect, which can be defined as the neglect of any type of stimuli (such as visual, tactile, auditory or mental representations) from the side opposite the brain lesion or a lack of spontaneous movement of the contralesional side of the body (or any part of the body toward the contralesional side), despite the ability to do so (2, 3). Prevalence estimates of neglect range from 25 to 80% of stroke survivors (4–7), depending on the methods of assessment, stage of recovery and type of neglect (8, 9). Neglect is associated with poorer functional outcomes such as reduced independence in daily tasks, higher risk of falls, longer length of hospital stays, and reduced likelihood of home discharge (10, 11). It is essential to accurately identify the presence of neglect, to reduce the burden for individuals, their careers and the health system (12).

However, the identification of neglect can be challenging. Neglect manifestations are heterogeneous and no single test can accurately identify all types of neglect (13, 14). Additionally, neglect presents on a scale from very severe to mild (15), with mild neglect only becoming noticeable in certain situations (16) such as navigating a busy environment. Many current assessment tools can lack the sensitivity to detect mild or moderate neglect (14, 17–20) resulting in people with undiagnosed neglect.

Multiple taxonomies for how neglect is defined and categorized into subtypes have been proposed [for example, see (2, 21, 22)]. The taxonomy recently mentioned (21) to capture and classify the heterogenous behaviors of neglect into three dimensions will be used in this review. The three dimensions of neglect include: (1) reference frame (as either egocentric or allocentric); (2) processing stage which includes perceptual subtypes (visual, tactile or auditory), representational, and motor; and (3) spatial sector (as either personal, peripersonal, and extrapersonal) (21). Neglect can be multimodal and occur in any or all combinations of the three dimensions (12), which makes assessment so challenging.

It is important to identify the subtype(s) of neglect for several reasons. Firstly, clinicians will be able to predict which daily tasks are impacted by the neglect behavior if the subtype is known. For example, visual neglect present in the far extrapersonal space will impact the ability to cross a road safely, however tasks such as reading, shaving or putting on make-up will not be impacted. The clinician will therefore be able to educate the person to increase their awareness of the risks associated with specific tasks and target interventions to compensate for the symptoms of neglect and improve the person's independence. If neglect in a particular subtype is not detected, the person may remain unaware of the risks associated with returning to their daily routines, potentially putting themselves and others at risk when attempting particular activities such as cooking, driving or crossing a road (19, 23).

Secondly, recovery from neglect may depend on the subtype. More people recover completely after 6 months from personal and extrapersonal neglect in comparison to peripersonal neglect (24). Also, some interventions may be more effective for particular neglect subtypes in comparison to others. For example, visual scanning training in isolation has been shown to improve visual related tasks such as reading with minimal effect on non-visual neglect behavior (25). Prism adaptation has shown to improve motor neglect, with no effect on perceptual neglect (26), while monocular patching may have the opposite effect on the two subtypes (27). Also cold water caloric stimulation might be more effective for sensory neglect in comparison to motor neglect (28). Consequently, the results of intervention studies that do not assess neglect subtypes may be invalid, indicating a combination of improvement in one subtype and no change of another type (29).

Neglect subtypes are referred to inconsistently across the literature. There are five systematic reviews on the assessment of neglect according to our knowledge (8, 30–33), however, none of them have considered assessments for all neglect subtypes across the three dimensions proposed.

A gold standard tool to identify all types of neglect does not exist (8, 13). Therefore, there is a need to identify all the tools available for each neglect subtype; identify the most robust tools; and develop a consensus on the most appropriate battery of tools for the diagnosis of neglect. This scoping review is the first stage of this process. Scoping reviews are used to map the available evidence across multiple disciplines, clarify the conceptual boundaries of a topic, key concepts and working definitions (34–36). In accordance with these guidelines, the objectives of this scoping review are to capture the reported working definitions for the subtypes of neglect and map the range of tools employed for assessing each neglect subtype.

The questions and sub questions we will answer with this scoping review are:

1. What are the neglect subtypes and their definitions reported in the literature?
2. What are the reported tools for assessing each identified subtype of neglect?

- What subtypes do the tools measure? (as explicitly stated in the included studies and also analyzed by the researchers)
- How has tool development changed overtime?

## Methods

This scoping review followed the guidelines for reporting outlined in the PRISMA extension for scoping reviews (PRISMA-Scr) (36). The study protocol is registered on the Open Science Framework (https://osf.io/bzv9q/). Any deviations from the protocol are described at the end of the paper.

The study selection criteria are outlined in Table 1. Inclusion criteria under each heading of Participants, Concept and Context were used to determine eligibility for inclusion in accordance with the guidelines for conducting scoping reviews (34, 35).

**Table 1:** Study selection criteria.

	**Inclusion criteria**	**Exclusion criteria**
Participants	Stroke	Neglect resulting from any other condition
	Adults, 18 years and older	Healthy controls
Concept	Any assessment of spatial neglect, or subtype of neglect that has some aspect of validity or reliability documented	Cognitive or perceptual assessment with no subtest for neglect
Context	No limitation. Assessment of neglect in any setting/stage post stroke. All primary research in any language	All secondary research

**Figure 1:**
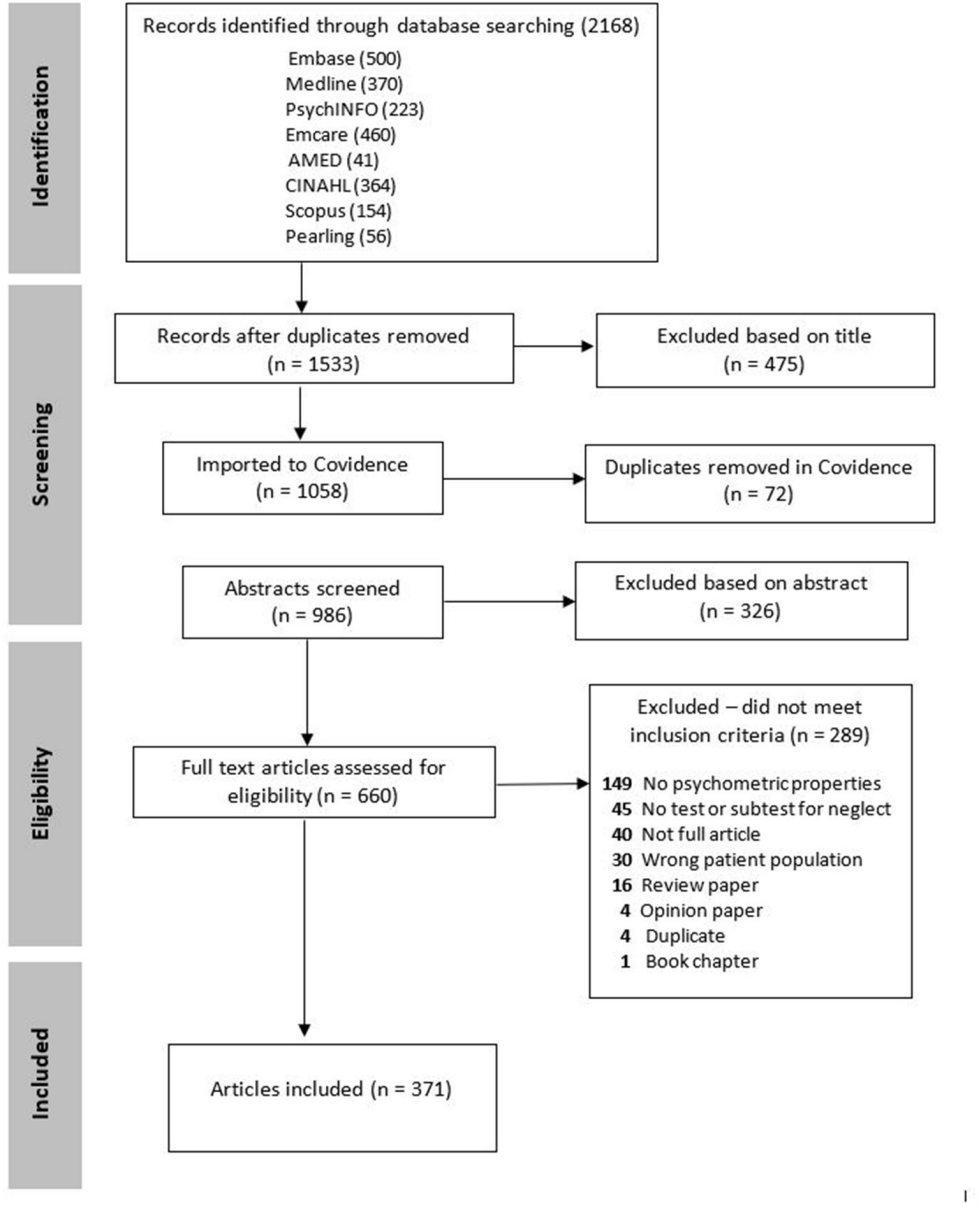
PRISMA screening flowchart of included studies.

The following databases were searched on 17th July 2020 from database inception: EMBASE (EMBASE, RRID:SCR_001650), Emcare, Medline (MEDLINE, RRID:SCR_002185) and psychINFO. Additionally, AMED and CINAHL databases were searched in January 2018, however they were not accessible to the authors in 2020 when completing the updated search. The search strategy was developed in conjunction with a research librarian. Search terms included combinations of the following terms: Stroke, CVA, cerebrovascular accident; and unilateral spatial neglect, spatial neglect, visual neglect, visual inattention; and all allied terms of neglect, assessment, evaluation, measurement, screening tools, psychometric properties. Full details of the database search strategy are available in the Supplementary Table S1. The top 10 authors working in neglect based on number of publications were also searched according to their citation indexes using Scopus to confirm that all relevant publications were obtained. The reference lists of all secondary research identified by the search strategy and the final included studies were hand searched for any additional relevant studies. Refer to Figure 1 for the PRISMA flow diagram of included studies.

The references identified from each database were imported into Endnote X8.2 (EndNote, RRID:SCR_014001). The duplicates were removed in Endnote and the titles were screened by one author for the obviously irrelevant studies before the remaining studies were exported into Covidence (Covidence, RRID:SCR_016484) (37). Two authors independently screened the titles and abstracts for eligibility for inclusion. The full texts of potentially eligible studies were retrieved and assessed for eligibility independently by two authors. Any discrepancies over the eligibility of studies were resolved through discussion with a third author.

The following information was extracted from each study: neglect subtype(s) and definition(s); name of tool(s); abbreviated name; description of tool and protocols followed; purpose (what does each tool purport to measure)?; population; primary author; country of residence; and discipline according to the primary author's affiliation. If the discipline was not evident from the listed affiliation, then a google search was completed (primary author's name and affiliation) and the discipline was recorded as per their current work webpage if available. Extracting the data and documenting the results was an iterative process. The extraction form was trialed on ten studies with two independent researchers as recommended by The Joanna Briggs Institute (35). The extracted data from the ten studies was discussed with the research team and the form was refined to ensure that all the relevant information was extracted. (For example, the neglect subtypes were not explicitly stated for most tools. Therefore, all the documented protocols for each tool were collated in more detail so that the analysis of the underlying subtypes could be completed by the research team).

All the terms used within the studies to label neglect and each neglect subtype were collated and presented in figures to display the frequency of terms used for each discipline. The documented definitions for each neglect subtype were collated, grouped and summarized. All neglect tools with documented protocols used in the included studies were collated. The description of each neglect tool and the documented protocols were analyzed by two authors to determine the underlying subtypes of each tool. Disagreements were discussed with the authorship team until an agreement was determined.

## Results

The systematic search produced 2,168 articles to be screened. After reviewing the titles and abstracts, the full texts of 660 articles were further reviewed for eligibility, resulting in 371 articles in the final sample for this review (see Figure 1 for details and full list of exclusion reasons). The included studies were published by a cross section of disciplines as listed in Table 2.

**Table 2:** Discipline of first author of included studies.

**Discipline**	**Total count**	**%**
Neurology	92	24.79
Psychology	90	24.25
Medicine / physical medicine & rehab	54	14.55
Occupational therapy	37	9.97
Neuropsychology	36	9.70
Cognitive and clinical neuroscience	27	7.28
Physiotherapy	12	3.23
Speech pathology	3	0.80
Orthoptics	2	0.54
Psychiatry	2	0.54
Development and education	2	0.54
Human movement	2	0.54
Bioengineering/biomechanics	2	0.54
Other	10	2.70

**Figure 2:**
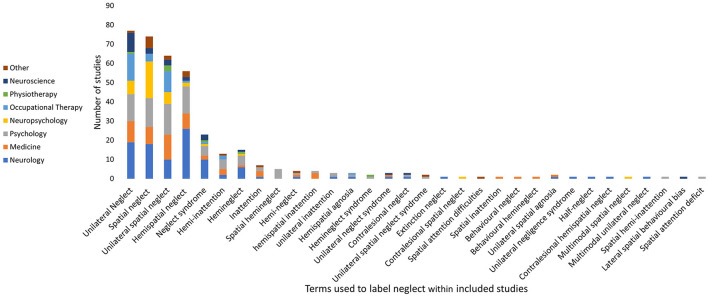
Neglect terms used within included studies.

To determine the reported definitions in the included studies, first the terms used to label neglect and the subtypes were collated, and summarized. There was no agreement on the terms used to refer to this disorder with 33 different terms used in the included studies (see Figure 2). The term *neglect* (excluded from Figure 2) was the most frequently used (174 studies, 47.3%). However, only 23 studies (6.2%) used the term *neglect* in isolation, with the majority of studies using it within the article as a shortened version of another, more descriptive term. There was variability across the disciplines with neurology having a clear preference for the term *hemispatial neglect*, however this was not consistent with other disciplines, such as neuropsychology preferring the term *spatial neglect*, while psychology was evenly split across the top four terms. The definitions for each neglect subtype were grouped according to the three dimensions of neglect (21).

### Dimension of Processing Stage

**Figure 3:**
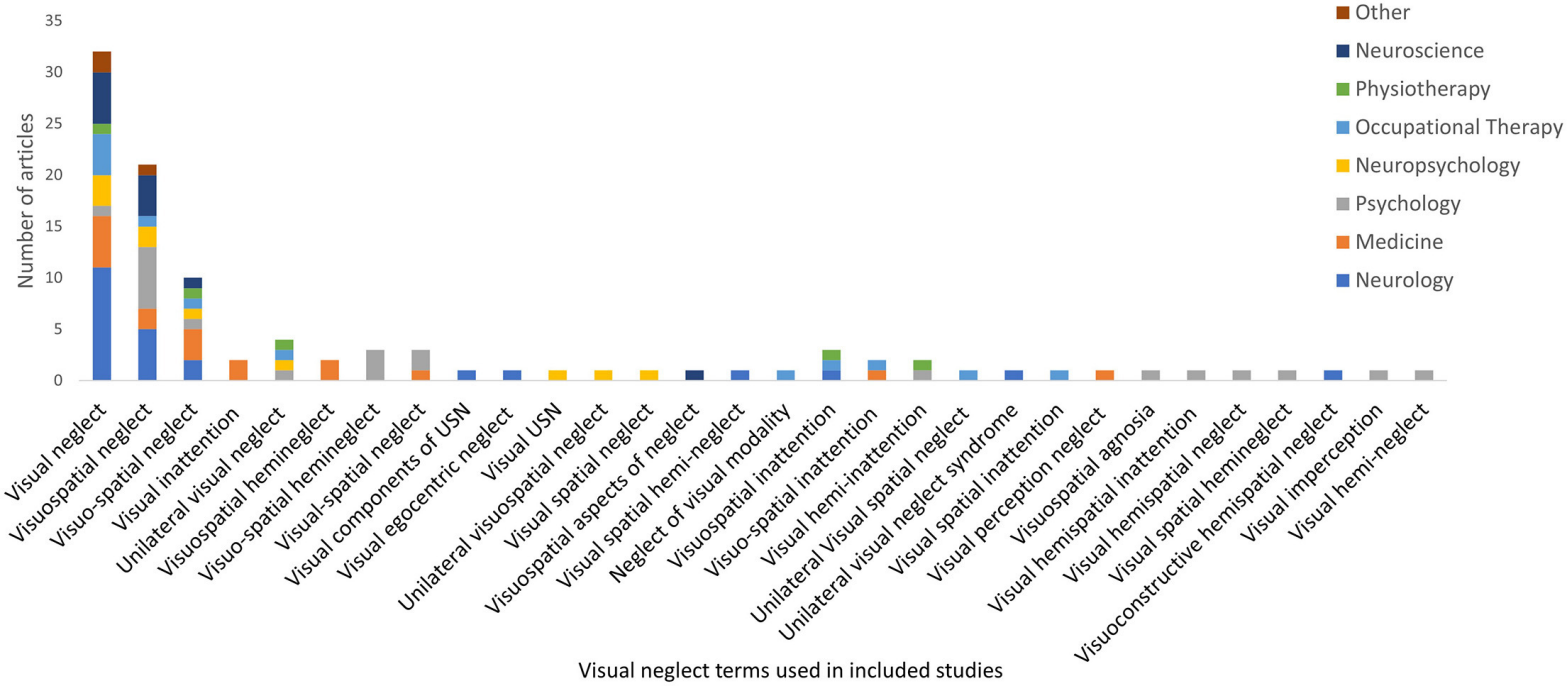
Visual neglect terms used within included studies.

**Figure 4:**
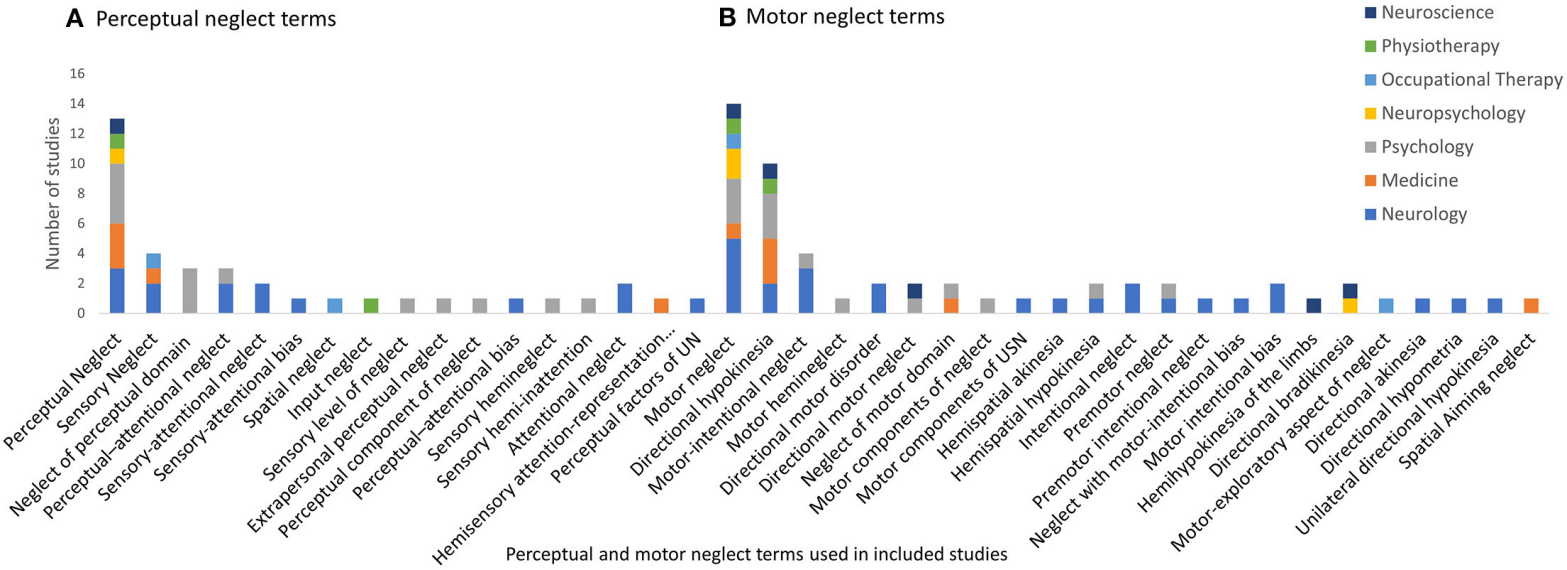
Perceptual and motor neglect terms used within included studies. **(A)** Perceptual and **(B)** Motor.

**Figure 5:**
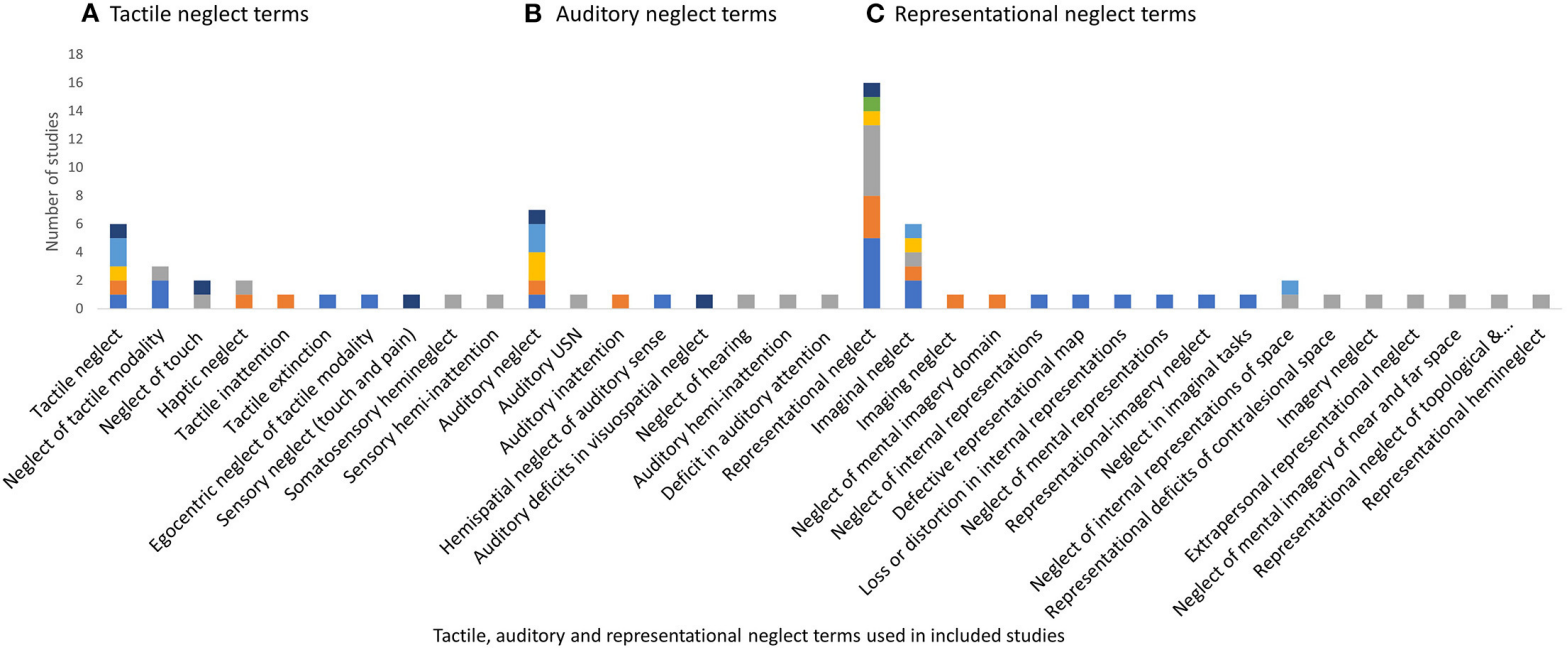
Tactile, auditory, and representational neglect terms used within included studies. **(A)** Tactile, **(B)** auditory, and **(C)** representational.

There were 105 different labels used for neglect subtypes under the *dimension of processing stage*, as displayed in Figures 3–**5**. These terms were grouped and labeled into the subtypes of perceptual (which includes the subtypes of visual, tactile and auditory), representational and, motor.

*Perceptual neglect* was reported in 38 studies with 17 different labels (Refer to Figure 4). The definitions varied considerably with some very broad definitions such as an impaired ability/failure to attend to or perceive stimuli in the contralesional hemispace (38, 39), while others specified the type of stimuli as including visual, auditory or tactile stimuli (40) or including both sensory/visuospatial and representational (mental imagery) aspects (41, 42).

*Visual neglect* was the most frequently reported subtype (101 studies, 27.4%), with 30 different terms used to describe it. The term *visual neglect* (see Figure 3) was preferred by all disciplines, apart from psychology who preferred the term *visuospatial neglect*. Although visual neglect was the most frequently reported subtype, the definitions still varied with the majority of studies either not defining it, or reporting a broad definition of neglect without defining the visual neglect subtype [for example (43, 44)], while few studies explicitly defined it as neglecting “visual stimuli” [for example (45–47)].

*Tactile neglect* was referred to in 20 studies with 10 different labels. The label of *tactile neglect* was the most frequently used (30%) (Refer to Figure 5). The majority of studies did not specify the type of tactile stimuli included in this subtype, describing it simply as neglect in the tactile modality (42, 48–51). Only two studies specified the type of tactile stimuli as somatosensory stimuli, including touch, pain and sensation (47, 52). Two studies additionally described tactile neglect as only occurring within an egocentric reference frame (53, 54), with a gradient of tactile neglect behaviors that increases further toward the contralesional side and gradually decreases over egocentric space toward the ipsilesional side.

*Auditory neglect* had *eight* different labels (Refer to Figure 5). A*uditory neglect* was the most frequently used label (47%) with the majority of the studies describing it simply as “neglect of the auditory sense” (45, 49, 55, 56). The behaviors of auditory neglect were described by Pavani, Làdavas & Driver (p. 181) (57) as a failure “to detect or identify contralesional sounds under bilateral presentation”, and may include poor position discrimination of contralesional sounds and an ipsilesional bias when pointing to contralesional sounds. Zimmer, Lewald & Karnath (58) proposed from the results of their study that auditory neglect in the strictest sense, described as neglect of contralesional auditory stimuli, even with no concurrent stimuli on the ipsilesional side, does not appear to exist.

*Representational neglect* had 17 different labels across 38 studies. The label of *representational neglect* was the most frequent (*n* = 16, 42.1%), followed by *imaginal neglect* (*n* = 6, 15.8%), see Figure 5. Representational neglect was described as the neglect of internal representations/mental images (40, 59–63) loss or distortion of mental images (64, 65); or an inability to build or explore the contralesional side of internal representations (64, 66). An impairment of spatial memory has also been implied due to the inability to retrieve portions of remembered scenes (39, 67, 68). On the other hand, representational neglect has been further categorized into (1) neglect of near static objects/locations and (2) neglect of far (topological) images (69, 70). It has also been suggested that representational deficits impact the ability to attend to or explore external contralesional space (71, 72), thus impacting on the execution of all daily tasks.

*Motor neglect* was identified with 23 different labels across 54 studies. *Motor neglect* was the most frequently used label (25.9%), followed by *directional hypokinesia*, (18.5%), see Figure 4. The descriptions of this subtype (*n* = 19) fell into two distinct categories as labeled by Bisiach et al. (73); (1) impaired spontaneous movement of the contralesional limb and (2) a directional specific deficit with impaired movement toward the contralesional side. The majority of studies considered motor neglect to incorporate behaviors from both categories (28, 38–40, 68, 74, 75). Other studies solely described a direction specific deficit (41, 42, 76–81). Although Bisiach and colleagues (73) described the direction specific deficit was irrespective of the limb being used, other studies considered this for the ipsilesional limb only (40, 75, 82).

### Dimension of Spatial Sector

**Figure 6:**
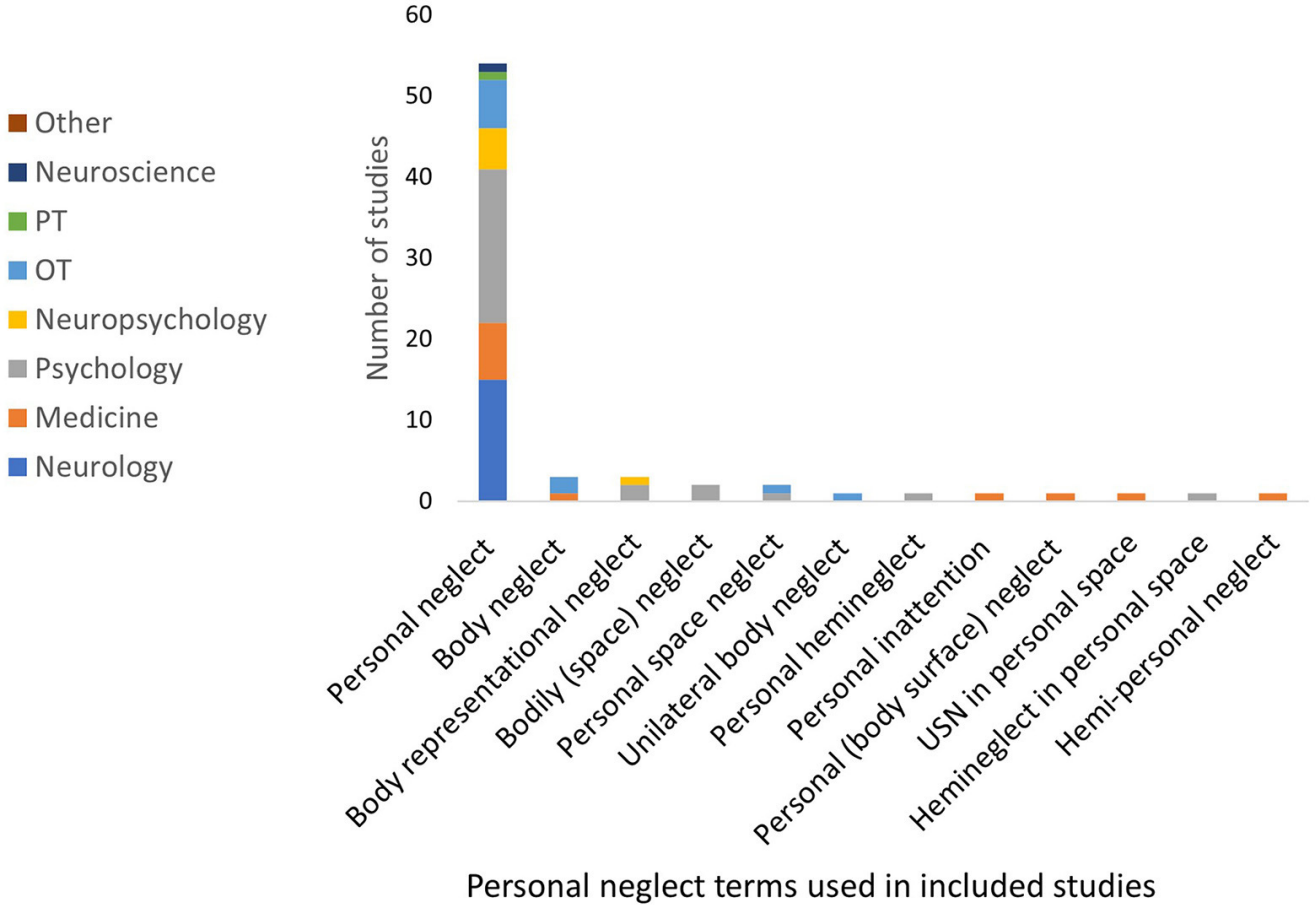
Personal neglect terms used within included studies.

*Personal neglect* had 12 different labels across 71 studies. The label of *personal neglect* was preferred by all disciplines (76%) (see Figure 6). Personal neglect was described as occurring *on the body* (*n* = 4); pertaining to the *body surface* (*n* = 5); occurring in *personal space* (*n* = 7); a lack of awareness (*n* = 5) or a *lack of exploration* (*n* = 3) of the contralesional side of the body. Alternatively, this subtype was also described by the impact on daily tasks (*n* = 4), such as neglecting to dress the left side of the body. The descriptions of this subtype were not consistent across all the studies, with six studies attributing this subtype of neglect to another underlying disorder such as representational neglect (having a disrupted representation of the contralesional side of the body) (83, 84), tactile (ignoring somatosensory stimuli) or motor neglect (underutilizing the contralesional side of the body) (84, 85), a disorder of the body schema (86, 87), or a disruption of the sense of ownership of the neglected side of the body (80, 86, 87).

**Figure 7:**
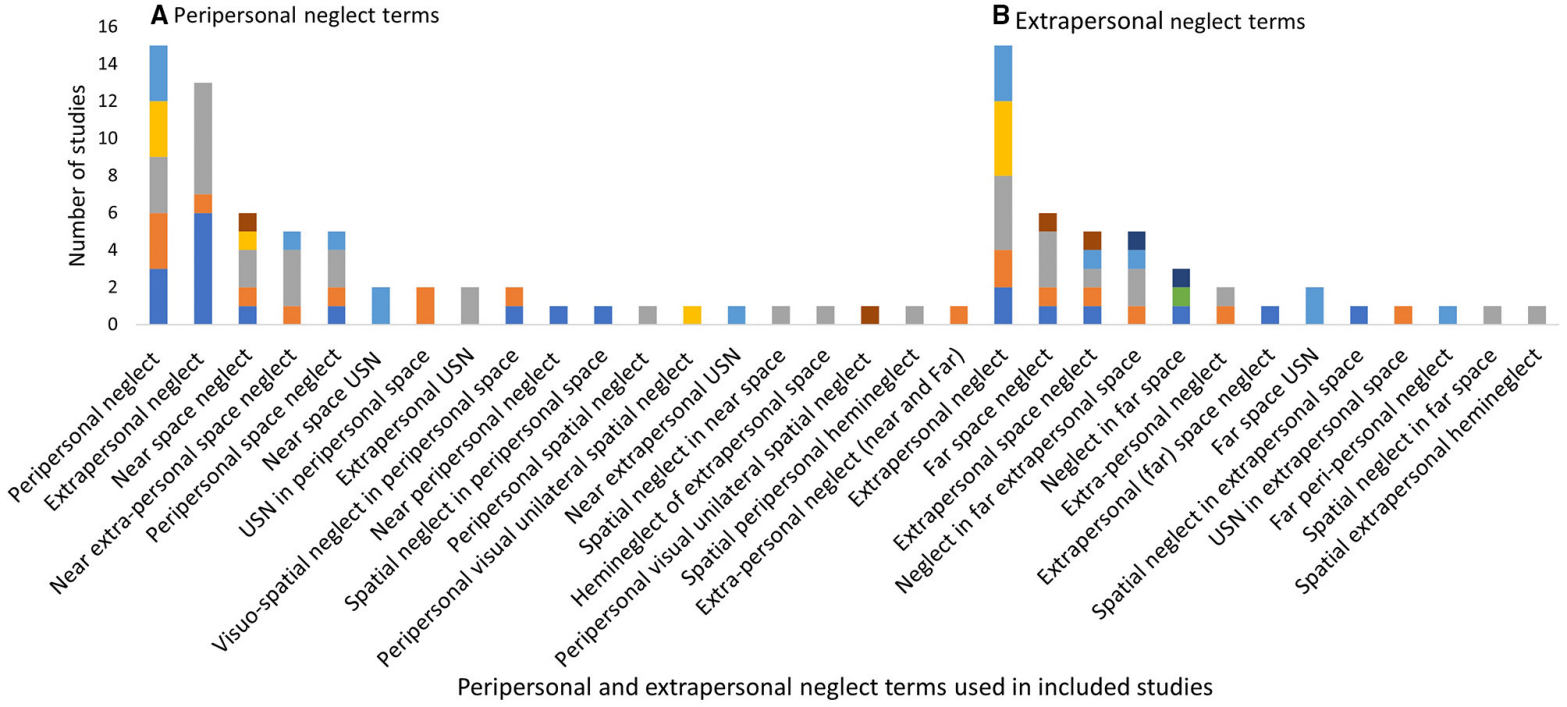
Peripersonal and extrapersonal neglect terms used within the included studies. **(A)** Peripersonal and **(B)** extrapersonal.

*Peripersonal neglect* was referred to in 62 studies with 19 different labels. P*eripersonal neglect* (*n* = 15, 24.2%) and *extrapersonal neglect* (*n* = 13, 21%) were most frequently reported, with no clear preference for either term (see Figure 7). The majority of studies described this subtype as neglect within arm's length or reaching distance (*n* = 17). Other studies simply stated this subtype as neglect within; the *peripersonal space* (*n* = 3), *near space* (*n* = 2), *near-extrapersonal space* (*n* = 2) or *space surrounding the body* (*n* = 6). This subtype was categorized more broadly by combining the neglect of near and far extrapersonal space into the one subtype of *extra-personal neglect*, or neglect of the environment (88). Conversely two studies further distinguished near extrapersonal space into further sub-subtypes of near radial (bottom half of an A4 page), far radial (top half of an A4 page), or diagonal neglect (neglect of the near left part of the page) (89, 90).

*Extrapersonal neglect* (neglect of the far space) had 13 different labels across 45 studies. The label of *extrapersonal neglect* was most frequently reported (*n* = 15) as displayed in Figure 7. The majority of studies (*n* = 19) described this subtype as “beyond reaching distance” (*n* = 19). Other descriptions include the neglect of the far space (70, 91, 92), or neglect of the navigational or walking space (93, 94).

### Dimension of Reference Frame

**Figure 8:**
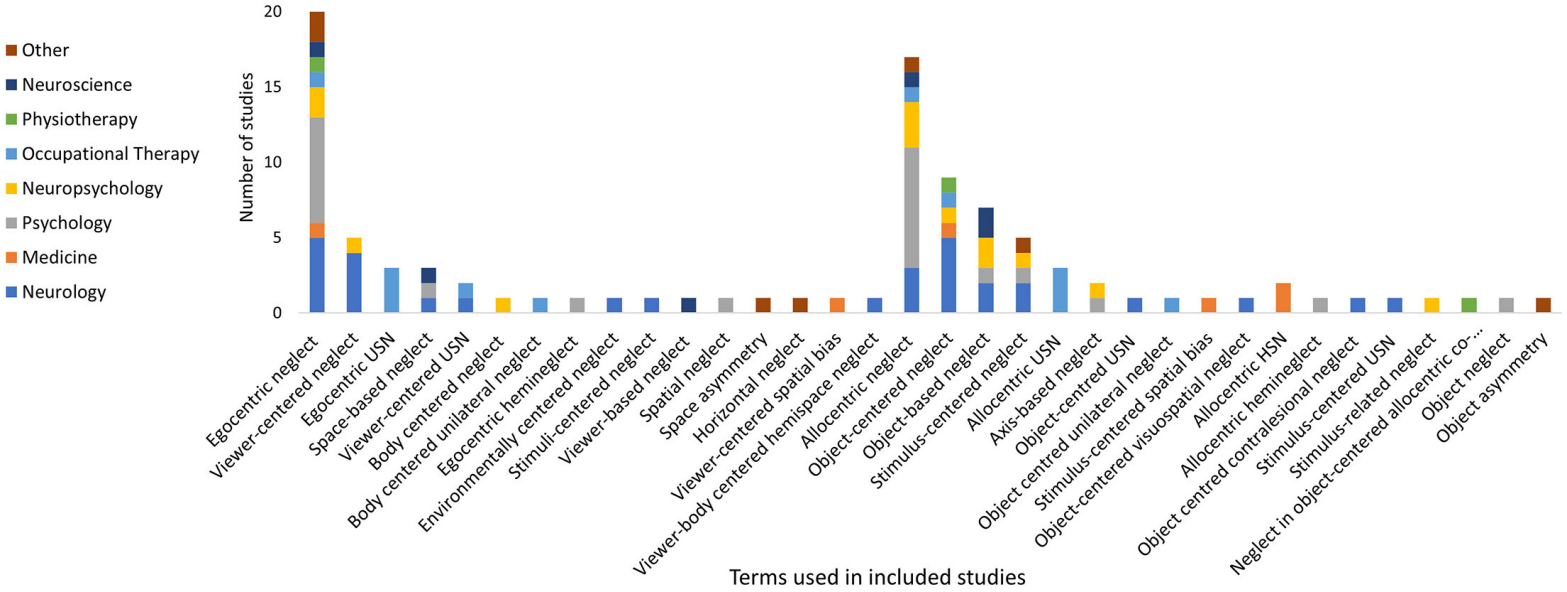
Egocentric and allocentric neglect terms used within included studies.

*Egocentric neglect* had 16 different labels within 44 studies. The label of *egocentric neglect* was the most frequently used (*n* = 20) (Refer to Figure 8). The majority of the studies described this subtype as egocentric (viewer-centered) neglect (*n* = 11), with some additionally describing that contralesional stimuli from a body centered/egocentric viewpoint are neglected (40, 55, 95, 96). Some descriptions reported the contralesional side of the spatial environment is neglected (97, 98), while others reported the contralesional side of the body is neglected (99, 100) or the “boundaries of the neglected space are not constant” (101).

*Allocentric Neglect* was referred to in 56 studies with 18 different labels (see Figure 8). The label of *allocentric neglect* was most frequently reported (*n* = 17). Descriptions of this subtype varied, as grouped into the following three main categories: (1) Neglect of the contralesional side of objects/stimuli irrespective of their location (53, 79, 98, 99, 102) commonly labeled as *stimulus-centered*. The side of the neglected object is determined by egocentric coordinates (103). (2) One side of objects are neglected regardless of their orientation (53, 79, 102, 104), commonly labeled as *object-centered*. For objects that have intrinsic left and right sides, the same side is neglected even if the object is mirror reversed, with the neglected side now being positioned on the ipsilesional side of the person (not influenced by egocentric coordinates). (3) Allocentric neglect is associated with egocentric neglect. It has been suggested that allocentric deficits are only observed in combination with egocentric neglect (97), or that the severity of allocentric deficits are influenced by egocentric factors (allocentric deficits are more severe toward the contralesional side) (95, 100, 105). Allocentric neglect has been challenged as a legitimate subtype of neglect, suggesting it is a particular form of egocentric neglect with the attentional window restricted to the individual object/stimuli (53, 103).

**Figure 9:**
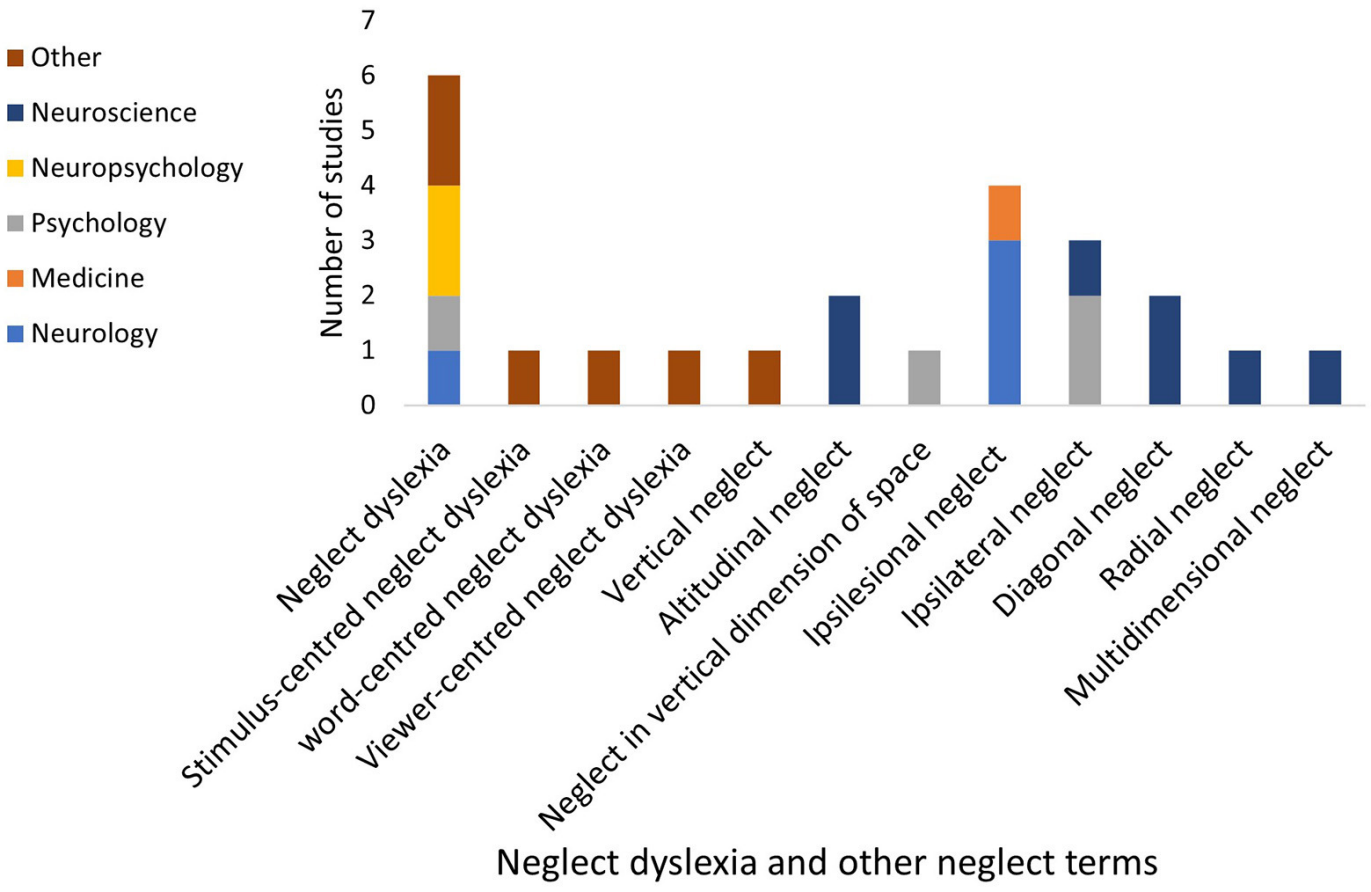
Neglect dyslexia and other neglect terms used within included studies.

Other neglect subtypes mentioned in the included studies, such as neglect dyslexia and ipsilesional neglect are displayed in Figure 9. Some of the studies considered extinction to be an aspect of spatial neglect [for example, (106, 107)] while others considered extinction to be a separate but related phenomenon [for example (52, 108)].

### Assessment Tools

This scoping review identified 292 tools for the screening or diagnosis of neglect. Most studies (*n* = 248, 67%) included a tool or battery of tools that did not specify the neglect subtype being assessed. The subtype investigated the most within the included studies, as explicitly stated by the authors was visual neglect (*n* = 90 studies, 24%), followed by personal neglect (*n* = 42, 11%) and peripersonal neglect (*n* = 38, 10%). Supplementary Table S2 in the supplementary material presents the names and references of all the spatial neglect tools and the analysis of the underlying subtypes that each tool identified. Most of the tools (57%) were unable to differentiate the underlying subtypes of visual or motor neglect contributing to deficient performance in a task. For example, the majority of the pen and paper tools could not determine if the left side of the paper was neglected due to the person not visually attending to that side of the paper or whether they were neglecting to move their arm toward that side. However, standardized tools that differentiate the neglect subtypes do exist. The number of tools that can identify each subtype is presented in Table 3.

**Table 3:** Standardized tools that can differentiate neglect subtypes.

**Neglect subtype**	**Number of tools**
Motor	9
Tactile	0
Visual	90
Auditory	6
Representational	9
Personal	12
Peripersonal space	216
Extrapersonal space	21
Egocentric	197
Allocentric	17

**Figure 10:**
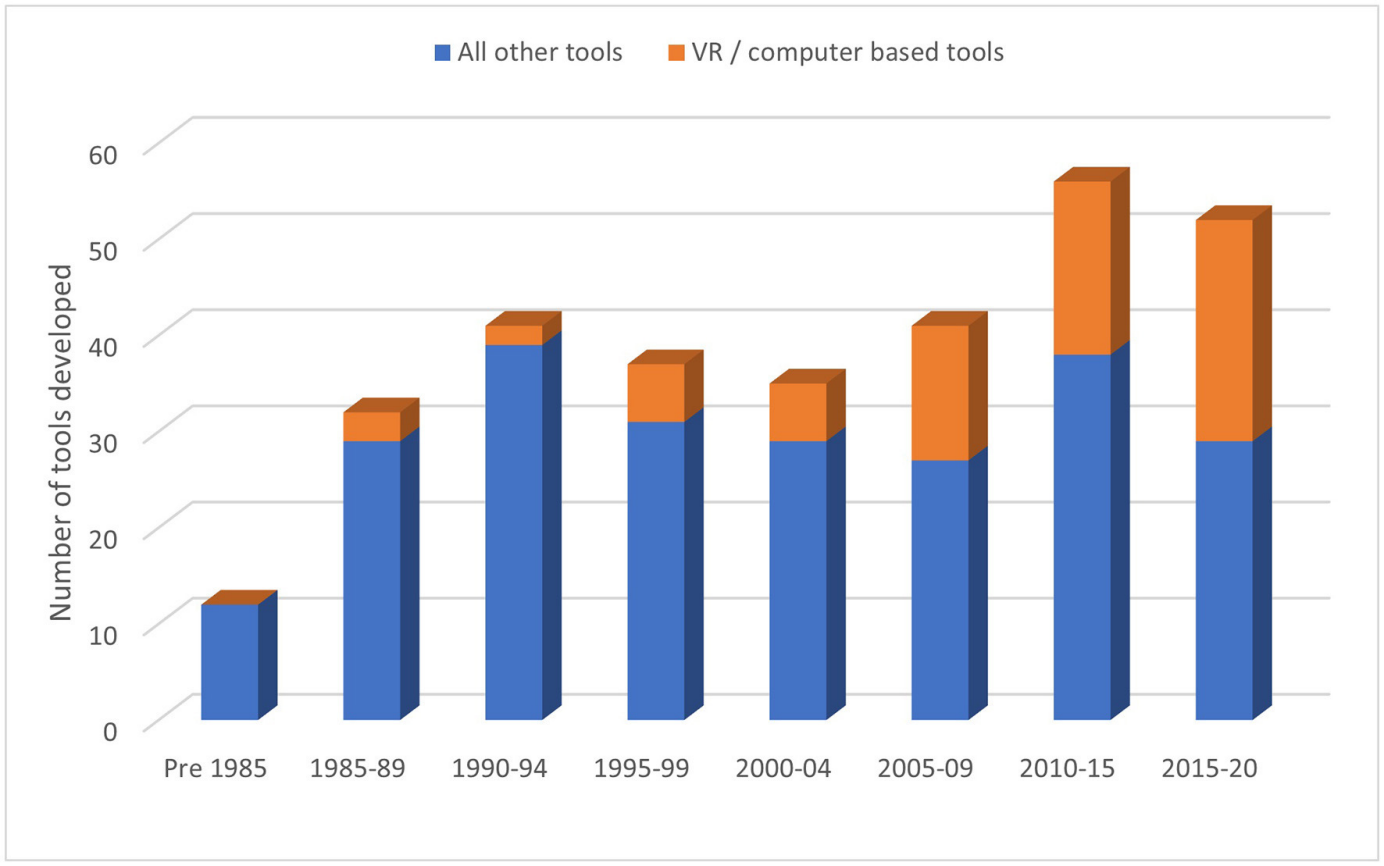
Number of virtual reality and computer-based tools.

The International Classification of Functioning, Disability and Health (ICF) categories that each tool evaluates are also listed in Supplementary Table S2. Most tools (88%) evaluated at the impairment level (in body function or structure), 18% activity limitations (difficulties in executing activities) and only 1.7% evaluated participation restrictions (difficulties with participating in life roles) (109). With no gold-standard tool for neglect, neglect tools are continually being developed, along with an increasing number of virtual reality and computer-based tools (see Figure 10).

## Discussion

The aim of this scoping review was to (1) collate the neglect subtypes and their definitions as reported in the literature and (2) to map the reported tools for assessing neglect and the identified subtypes.

It is evident from the results of this scoping review that there is a lot of variability with the terms used to label this syndrome as a whole and the individual subtypes. There is no consistency with the terms used for each subtype with some terms being used for multiple subtypes. For example, “extrapersonal” is sometimes used to refer to the reaching space (110), while many studies use this term to refer to the far space or outside of reaching (111–113). On the other hand, “extrapersonal” has also been used refer to neglect of the environment; encompassing both within and outside of reaching distance (80). Additionally, some subtypes are being described and grouped into different categories by different studies. For example, perceptual neglect is categorized as incorporating visual and representational neglect by some (41, 42), or visual, tactile and auditory by others (40). Some subtypes have multiple conflicting definitions such as personal neglect and allocentric neglect, which have both been challenged as legitimate neglect subtypes. This inconsistency with the terms and definitions used to refer to the neglect subtypes is creating confusion across the field and is a major barrier for clinicians to understand, compare and apply the literature to clinical practice.

Additionally, there is no agreement on the tools used for identifying neglect. This scoping review has revealed almost 300 neglect tools with documented protocols, and the development of new tools is not slowing down. The majority of tools are brief paper and pencil screening tools that are frequently used in combination with one or two other tools for screening, or as a suite of tools for the comprehensive diagnosis of neglect. Apart from a few validated batteries such as the Behavioral Inattention Test (114), many of the neglect batteries used in the included studies were made from different combinations of tools that have not been validated when used together as a diagnostic suite. This has resulted in high variability in the reported incidence of neglect and a plethora of research that cannot be effectively collated together.

It is important to identify the presenting neglect subtypes to assist with predicting and ameliorating the impact on daily life. Visual neglect in the peripersonal space is most commonly assessed, however the sheer number of tools available for these subtypes would be overwhelming for clinicians to navigate. Alternatively, very few standardized tools for tactile and representational neglect subtypes exist. Only one representational neglect tool (O'clock test) was identified in this review that is not dependent on knowledge of local landmarks and thus could be standardized for use in any country. However, this test has been found to be too difficult to complete for the majority of people with neglect (115). This may be due to the tool relying on other functions such as sustained attention and intact executive functions, which are often impaired after stroke. This can be said for many neglect tools, such as the mirror or pulley devices developed to dissociate motor and visual neglect [for example, see (71, 73, 116)], with impaired performance not necessarily reflecting true neglect behavior if executive functions are impaired. Gaps also exist for identifying motor or auditory neglect in clinical practice. Several tools exist for identifying both subtypes, however many of them are not feasible for use in clinical practice due to the individualized set up and equipment requirements.

Pen and paper tools are commonly used in clinical practice, however they only identify impairments in one spatial dimension—within peripersonal (reaching) space. The use of pen and paper tools may result in a person with undetected neglect in either personal or extrapersonal (outside of reaching) space. Another limitation with pen and paper tools is the inability to differentiate between motor and visual neglect as mentioned previously. Pen and paper tools may be useful to screen for neglect, however other methods of assessment need to be considered for the comprehensive diagnosis of all neglect behaviors.

Tools developed to tease apart the motor and perceptual aspects of neglect have not been able to categorize the two subtypes consistently (117, 118). It has been suggested that minor differences in task requirements such as reaching for a direct target vs. a spatial /delayed judgment may account for these discrepancies (119). Moreover, it is uncertain if neglect subtypes are transient or consistent over time. In the subacute phase post stroke, significant variability in the categorization of visual, motor and personal neglect was observed over three test sessions in 18 (86%) participants (120). The pattern of variability was not consistent with practice effects or spontaneous recovery. It is unclear whether these results can be explained by methodological limitations such as low test-retest reliability of the neglect tools, or actual fluctuations in the neglect behavior.

New tools for identifying neglect are continually being developed, possibly due to the existing tools not being able to detect milder forms of neglect, or an inability to detect the different neglect subtypes. The increasing number of virtual reality and computer-based tools being developed have the potential to increase the task demands, such as dual-task paradigms or speeded reaction time tests, in order to identify mild or subclinical neglect (121–123). However, many of these tools are not feasible to use in clinical settings due to the complex setup or cost requirements.

The following questions were raised by the research team during the process of mapping the subtypes identified with specific tools: (1) All tasks tap into eye movements—thus are all of them tapping into motor (oculomotor) aspects of neglect? It was decided that for the purpose of this review to indicate motor neglect only for the tasks that require a motor response, as opposed to a verbal response or the tracking of eye movements. And (2) If spatial neglect is due to an impairment of spatial representation (124, 125), then do all tasks that require a motor response also tap into representational neglect? For the purpose of this review only tasks that rely on visual memory, such as drawing objects from memory or completing tasks with the eyes closed, along with completing functional tasks in personal space were indicated as being influenced by representational neglect. However, these questions would benefit from further consideration in future studies.

### Limitations

We acknowledge that by limiting the included studies to ones that have documented some psychometrics of a tool, this may have excluded some tools from the full analysis. However, we did this to ensure at least some level of investigation of robustness in the tools included. Additionally, determination of discipline of the first author based on their affiliations and work webpage may not be an accurate reflection. However, contacting all the authors to confirm was outside the scope of this review.

## Conclusion

There was no consistency with the terms used to refer to neglect and presenting subtypes. It is essential to unify the terminology and definition for each subtype of neglect. There are hundreds of neglect tools available, however many are not able to differentiate presenting subtypes. There have been multiple tools developed to dissociate different types of neglect such as egocentric and allocentric neglect; however, the responsiveness, validity and reliability of these tools has not been compared. It is important for clinicians and researchers to critically evaluate the neglect tools being used for the screening and diagnosis of neglect. The results of this scoping review will inform the scope of tools to be included in a full systematic review summarizing the reliability, validity, responsiveness and utility of neglect tools used in clinical practice. We recommend further work to develop consensus around neglect subtypes, definitions, and assessment.

## Deviations From Protocol

This scoping review has not answered the following sub-questions as originally proposed in the protocol of (1) “what definitions have been used by different disciplines?”; and (2) “how have the definitions evolved over time?” There were relatively few studies that described each subtype so trends between the different disciplines or over time were unable to be made. The definitions for each subtype were analyzed collectively. Additionally, it was deemed outside the scope of this review to comment on “what tools are being used by different disciplines?” as originally proposed. It was decided that the included studies may not be representative of all the tools used across the disciplines as the criteria for inclusion were only studies that examined some aspect of validity or reliability of a tool.

## Data Availability Statement

The original contributions presented in the study are included in the article/Supplementary Material, further inquiries can be directed to the corresponding author.

## Author Contributions

LW completed the database searches and wrote the first draft of the manuscript. LW and TL contributed to the data extraction and analysis. All authors contributed to conception and design of the study, screening of abstracts and full texts, manuscript revision, and read and approved the submitted version.

## Conflict of Interest

The authors declare that the research was conducted in the absence of any commercial or financial relationships that could be construed as a potential conflict of interest.

## Publisher's Note

All claims expressed in this article are solely those of the authors and do not necessarily represent those of their affiliated organizations, or those of the publisher, the editors and the reviewers. Any product that may be evaluated in this article, or claim that may be made by its manufacturer, is not guaranteed or endorsed by the publisher.
