# Supplementary material for: Spatial Neglect Subtypes, Definitions and Assessment Tools: A Scoping Review
Source: Front Neurol. 2021 Nov 24;12:742365. doi: 10.3389/fneur.2021.742365 (PMC8653914; doi:10.3389/fneur.2021.742365)
Supplement: Supplementary file 1 [file Table_1.pdf]

Supplementary Material

Table S1. Search Strategy for Medline

1. cerebrovascular disorders/
2. exp basal ganglia cerebrovascular disease/
3. exp brain ischemia/
4. exp carotid artery diseases/
5. exp intracranial arterial diseases/
6. exp "intracranial embolism and thrombosis"/
7. exp intracranial hemorrhages/
8. stroke/
9. exp brain infarction/
10. stroke, lacunar/
11. vasospasm, intracranial/
12. vertebral artery disection/
13. stroke or poststroke or post-stroke or cerebrovasc* or brain vasc* or cerebral vasc* or cva* or apoplex* or SAH
14. (brain* or cerebr* or cerebell* or intracran* or intracerebral) adj3 (isch?emi* or infarct* or thrombo* or emboli* or occlus*)
15. (brain* or cerebr* or cerebell* or intracran* or intracerebral or subarachnoid) adj3 (haemorrhage* or hemorrhage* or haematoma* or hematoma* or bleed* or disease)
16. hemiplegia/
17. exp paresis/
18. hemipleg* or hemipar* or paresis or paretic
19. 1 or 2 or 3 or 4 or 5 or 6 or 7 or 8 or 9 or 10 or 11 or 12 or 13 or 14 or 15 or 16 or 17 or 18
20. perceptual disorders/
21. exp visual perception/
22. space perception/
23. attention/
24. hemineglect or hemi-neglect
25. (unilateral or spatial or hemi?spatial or visual or syndrome or tactile or auditory or motor) adj3 neglect
26. Visual inattention or sensory inattention or hemi-inattention or visual extinction or sensory extinction, or tactile extinction or auditory extinction
27. (perceptual or attention* or visuo?spatial or visuo?perceptual) adj3 (disorder* or deficit* or impairment* or inabilit* or problem)
28. 20 or 21 or 22 or 23 or 24 or 25 or 26 or 27
29. test or clinical measure* or assessment* or evaluation* or measurement* or screening tool* or outcome measure* or scale or instrument
30. "Sensitivity and Specificity"/
31. Psychometric properties or specificity or sensitivity or reliab* or valid* or clinimetric or diagnostic accuracy
32. 30 or 31
33. 19 and 28 and 29 and 32
34. (exp animals/ or exp invertebrate/ or animal experiment/ or animal model/ or animal tissue/ or animal cell/ or nonhuman/)
35. 33 not 34
36. limit 35 to (humans and "all adult (19 plus years)")
