# Supplementary material for: Spatial Neglect Subtypes, Definitions and Assessment Tools: A Scoping Review
Source: Front Neurol. 2021 Nov 24;12:742365. doi: 10.3389/fneur.2021.742365 (PMC8653914; doi:10.3389/fneur.2021.742365)
Supplement: Supplementary file 2 [file Table_2.pdf]

Supplementary Material

Table S2. Neglect screening and assessment tools with documented protocols - analysis of neglect subtypes and ICF categories

Tool name (reference)	Egocentric	Allocentric	Personal	Peripersonal	Extrapersonal	Representation	Visual	Tactile	Auditory	Motor	Impairment	Activity Limitation	Participation Restriction
Behavioural Inattention Test (BIT)[1] modified scoring [2]	✓	x		✓		x	✓			x	✓	✓	
BIT-cs (conventional subtests)	✓	x		✓		x	x			x	✓		
BIT-bs (behavioural subtest)	✓	x		✓			✓			x	✓	✓	
sBIT (3-item version) [3]	✓	x		✓			x			x	✓		
mBIT for acute stroke (8 item version) [4]	✓			✓	✓		✓			x	✓	✓	
Chinese Behavioural Inattention Test–Hong Kong version (CBIT-HK) [5]	✓	x		✓		x	✓			x	✓	✓	
BIT Japanese version [6,7]	✓	x		✓		x	✓			x	✓	✓	
BIT Hebrew version [8,9]	✓	x		✓		x	✓			x	✓	✓	
Neglect Test (NET) (Fels & Geissner, 1996) (German BIT)[10, 11]	✓	x		✓		x	✓			x	✓	✓	
BIT-E (Spanish version) [12]	✓	x		✓		x	✓			x	✓	✓	
Sunnybrook Neglect Assessment Procedure (SNAP)[13]	✓	x		✓			x			x	✓		
Batterie d'Evaluation de la Négligence (BEN) [14,15] (GEREN neglect battery)	✓	x		✓		x	✓			x	✓	✓	
Rivermead Perceptual Assessment Battery [16]	x	x		✓			x			x	✓		
Northwick Park Examination of Cognition [17] Perception component	✓	x		✓			x			x	✓		
Strokevision app [18]	✓	x		✓			x			x	✓		
Vision Impairment Screening Assessment (VISA) tool[19]	✓	x		✓			x			x	✓		
Screening Instrument for neuropsychological impairment in stroke (SINS)[20]	✓	x	x	x		x	x			x	✓		
Battery for the evaluation of hemineglect [21]	✓			✓			✓			x	✓	✓	
OT-APST (Adult Perceptual Screening test) neglect subtest [22]	x	x		✓		x	✓			x	✓	✓	
OSOT (Ontario Society of Occupational Therapists) Evaluation of Perceptual Function [23]	x	x		✓		x	x			x	✓		
The Ontario Society of Occupational Therapy (OSOT) Perceptual Evaluation French version [24] - Visual scanning and visuo-spatial neglect subtest	x	x		✓		x	x			x	✓		
The Visuospatial Neglect Test Battery [25]	✓	x		✓		x	x			x	✓		
Circle Monitor CM Tasks [10]	✓	x		✓			x			x	✓		

✓ Tool differentiates the neglect subtype.
x Deficient performance may be attributable to the subtype, but the scoring does not allow for it to be differentiated from other subtypes.
? Unable to determine from documented protocols.
(x) Alternative protocols of the test involve this subtype (for example motor response if unable to verbalise response)

Tool name (reference)	Egocentric	Allocentric	Personal	Peripersonal	Extrapersonal	Representation	Visual	Tactile	Auditory	Motor	Impairment	Activity Limitation	Participation Restriction
**Cancellation tasks**													
Bell's test[26] (modified protocols [27,28])	✓			✓			X			X	✓		
Star cancellation test [1]	✓			✓			X			X	✓		
Star cancellation test - Russion version [29]	✓			✓			X			X	✓		
line cancellation test [1], Albert's Test[30,31] (40 lines)	✓			✓			X			X	✓		
Mesulam shape cancellation task [32]	✓			✓			X			X	✓		
Line cancellation task 30 x short lines [33,34]	✓			✓			X			X	✓		
Line cancellation (Left, middle and right of midline)[35]	✓			✓			X			X	✓		
Barrage test/ Line cancellation test) [21] (21 lines, A3 page)	✓			✓			X			X	✓		
Ellipses cancellation task [36]	✓			✓			X			X	✓		
BIT - short screening test [37] (2 items: Letter & star)	✓			✓			X			X	✓		
BIT cancellation tasks (letter, line, star)	✓			✓			X			X	✓		
Computerised cancellation tasks [38]	✓			✓		X	X			X	✓		
Balloons test [39]	✓			✓			X			X	✓		
Digitized shape cancellation task [40]	✓			✓			X			X	✓		
Behavioural task [34]	✓			✓			X			X	✓		
Head mounted display (HMD) - line & star cancellation[6]	✓	x		✓			X			X	✓		
VR and haptics cancellation task [41]	✓			✓			X			X	✓		
Touch screen cancellation tasks) (letter and shape) [42]	✓			✓			X			X	✓		
Letter cancellation task (SLCT/H cancellation Task) [43,44]	✓			✓			X			X	✓		
(Random) Letter cancellation task [32,45]	✓			✓			X			X	✓		
Double Letter Cancellation Test (DLCT) [44,46]	✓			✓			X			X	✓		
Letter cancellation test [47]	✓			✓			X			X	✓		
O/X cancellation task with graphics tablet [48]	✓			✓			X			X	✓		
Structured Chinese Word Cancellation Test (SCWCT) [49]	✓			✓			X			X	✓		
Random Chinese Word Cancellation Test (RCWCT) [49–51]	✓			✓			X			X	✓		
Custom cancellation test "T" [52]	✓			✓			X			X	✓		
Letter cancellation (BIT subtest)[1] (timed version [28])	✓			✓			X			X	✓		
O cancellation test (visible/invisible marks) [53]	✓			✓			X			X	✓		
Letter cancellation test [54]	✓			✓			X			X	✓		

✓ Tool differentiates the neglect subtype.
x Deficient performance may be attributable to the subtype, but the scoring does not allow for it to be differentiated from other subtypes.
? Unable to determine from documented protocols.
(x) Alternative protocols of the test involve this subtype (for example motor response if unable to verbalise response)

Tool name (reference)	Egocentric	Allocentric	Personal	Peripersonal	Extrapersonal	Representation	Visual	Tactile	Auditory	Motor	Impairment	Activity Limitation	Participation Restriction
**Egocentric and allocentric cancellation tasks**													
Apples cancellation test [55]	✓	✓		✓			x			x	✓		
Birmingham Cognitive Screen (BCoS) [56]	✓	✓		✓			x			x	✓		
Hong Kong Birmingham Cognitive Screen (HKBCOS) [57]	✓	✓		✓			x			x	✓		
Cantonese Birmingham Cognitive Screen (Cantonese BCoS) [58]	✓	✓		✓			x			x	✓		
Oxford Cognitive Screen: Broken hearts cancellation task [59]	✓	✓		✓			x			x	✓		
Hong Kong version of Oxford Cognitive Screen: Broken hearts cancellation task (HK-OCS) [60]	✓	✓		✓			x			x	✓		
Russion Version of the Oxford Cognitive Screen (Rus-OCS) [61]	✓	✓		✓			x			x	✓		
3S Spreadsheet Test (3S Test) [62]	✓	✓		✓			x			x	✓		
Ota circle cancellation [63] (Gap detection task, Ota search task)	✓	✓		✓			x			x	✓		
modified Gap Detection Test [64] (Visual and Tactile)	✓	✓		✓			x	x		x	✓		
Defect detection task - circle and triangle [63]	✓	✓		✓			x			x	✓		
Apples cancellation test Italian standardization [55,65]	✓	✓		✓			x			x	✓		
**Motor tasks**													
Lateralised motor performance [66]	✓		✓							✓	✓		
Computerised line bisection Task - Fractionated where and aiming measures [66]	x	x		✓			✓			✓	✓		
Closed circuit camera of line bisection and cancellation tasks [67]	✓			✓			✓			✓	✓		
Paradigm for assessing directional motor bias [68]	✓			✓			✓			✓	✓		
Motor and perceptual RT task [69]	✓			✓			✓			✓	✓		
Landmark line bisection task (Landmark Test) [70]	✓			✓			✓			✓	✓		
Modified landmark task (mLMT) [71]	x	x		✓			✓			✓	✓		
Congruent and non-congruent bisection task [72]	x	x		✓			✓			✓	✓		
Wooden handle [73]	✓			✓						✓	✓		
Exploratory-motor test [74]	✓			✓		x				x	✓		

✓ Tool differentiates the neglect subtype.
x Deficient performance may be attributable to the subtype, but the scoring does not allow for it to be differentiated from other subtypes.
? Unable to determine from documented protocols.
(x) Alternative protocols of the test involve this subtype (for example motor response if unable to verbalise response)

Tool name (reference)	Egocentric	Allocentric	Personal	Peripersonal	Extrapersonal	Representation	Visual	Tactile	Auditory	Motor	Impairment	Activity Limitation	Participation Restriction
**Line bisection tasks**													
Schenkenberg Line bisection task (modified scoring [75])	x	x		✓			x			x	✓		
Line bisection - (2 x 10cm, 2 x 15cm, 2 x 25cm lines) [76]	x	x		✓			x			x	✓		
Line bisection test (BIT)[1] (timed version [28])	x	x		✓			x			x	✓		
Line bisection [77] (24 x 20cm)	x	x		✓			x			x	✓		
Line bisection test (3 x 16cm and 3 x 24cm) [78]	x	x		✓			x			x	✓		
Line bisection (2 x 5cm and 2 x 20cm) [14] (% deviation)	x	x		✓			x			x	✓		
Line bisection task (5 x 6cm, 5 x 7cm, 5 x 8cm [79]	x	x		✓			x			x	✓		
Line bisection task 3 x 20cm line [33]	x	x		✓			x			x	✓		
Solid line bisection task (10 x 24.2cm) [80,81]	x	x		✓			x			x	✓		
Line bisection 5 x 24cm [82,83]	x	x		✓			x			x	✓		
Line bisection (3 x 17cm lines) [84]	x	x		✓			x			x	✓		
Line bisection (3 x 22cm lines on A4 page) [85]	x	x		✓			x			x	✓		
Line bisection task (5 x 18cm) [86]	x	x		✓			x			x	✓		
Line bisection (27cm) (page to the L, R and midline) [87]	x	x		✓			x			x	✓		
Complex line bisection test [88,89]	x	x		✓			x			x	✓		
Line bisection [90] (36 lines 26cm/30cm (numbers at either end, presented left and right of midline)	x	x		✓			x			x	✓		
Stroke Impairment Assessment Set (SIAS) - visuospatial perception subtest [91,92] (Bisect 50cm tape x 2)	x	x		✓			x			x	✓		
Line bisection test (10 x 10.7cm) [54]	x	x		✓			x			x	✓		
Line bisection task (5 x 18cm) [36]	x	x		✓			x			x	✓		
Horizontal line bisection [93]	x	x		✓			x			x	✓		
Line bisection task (5 x 242mm) [94]	x	x		✓			x			x	✓		
Line bisection (6 lines on page offset from centre) [18]	x	x		✓			x			x	✓		
Line bisection 25 x 15 cm [95]	x	x		✓			x			x	✓		
Line bisection (10 x 24cm: 5 left of midline, 5 right of midline) [96]	x	x		✓			x			x	✓		
Bisection of a 20cm horizontal line [97]	x	x		✓			x			x	✓		

✓ Tool differentiates the neglect subtype.
x Deficient performance may be attributable to the subtype, but the scoring does not allow for it to be differentiated from other subtypes.
? Unable to determine from documented protocols.
(x) Alternative protocols of the test involve this subtype (for example motor response if unable to verbalise response)

Tool name (reference)	Egocentric	Allocentric	Personal	Peripersonal	Extrapersonal	Representation	Visual	Tactile	Auditory	Motor	Impairment	Activity Limitation	Participation Restriction
**Line bisection tasks (continued)**													
Line bisection (8 lines 3 x 6.2cm, 3 x 10cm, 2 x 18cm, cumulative % deviation) [98]	x	x		✓			x			x	✓		
Character Line Bisection Task (CLBT: Letter-line and Star-line Bisection Tasks) [80]	x	x		✓			x			x	✓		
Computerized touch screen line bisection test (300 x (20cm, 25cm, 30cm lines) (in middle, L, R space with/o distractions) [99]	x	x		✓			x			x	✓		
Rebisection task [100]	x	x		✓			x			x	✓		
Line quadrisection task [101]	x	x		✓			x			x	✓		
Line extension task (5x6cm, 5x7cm, 5x8cm in centre of A4 page) [79]	x	x		✓			✓			✓	✓		
L figure line extension task [102]	x	x		✓			x			x	✓		
Bisection of Judd stimuli (10cm and 12cm rods with visual, haptic, and visuo-haptic presentation) [103]	x	x		✓		x	x	x		x	✓		
Extended Line Bisection task (LB) [104,105]	x	x		✓			x			x	✓		
**Pointing/grasping objects**													
Pointing/grasping centre of rod [106]	x	x		✓			x			x	✓		
Baking Tray Task (BTT) (Tham & Tegner) [3,107]	✓			✓			x			x	✓		
Baking Tray Task with E-TAN (E-BTT) [108]	✓			✓			x			x	✓		
modified version of Baking Tray Task [109]	✓			✓			x			x	✓		
Table Test (Kerkhoff) [52]	✓			✓			x			x	✓		
Visual search board (VSB; Kimura) [13]	✓			✓			x			x	✓		
Robotic object hitting task [110]	✓			✓			x			x	✓		
Two-part picture test [111]	✓			✓			x			x	✓		
Object-finding test [89]	✓			✓			x			x	✓		
The shape test [112]	✓			✓		x	x	x		x	✓		

✓ Tool differentiates the neglect subtype.
x Deficient performance may be attributable to the subtype, but the scoring does not allow for it to be differentiated from other subtypes.
? Unable to determine from documented protocols.
(x) Alternative protocols of the test involve this subtype (for example motor response if unable to verbalise response)

Tool name (reference)	Egocentric	Allocentric	Personal	Peripersonal	Extrapersonal	Representation	Visual	Tactile	Auditory	Motor	Impairment	Activity Limitation	Participation Restriction
**Copying tasks**													
5-element complex drawing (a house and four trees) (Gainotti drawing, Landscape drawing) [113]	x	x		✓			x			x	✓		
Copying Task (a fence, a car, a house and a tree) [114]	x	x		✓			x			x	✓		
Ogden Scene (tree, fence, house, tree) [115](landscape drawing, scene/ figure copy) [14]	x	x		✓			x			x	✓		
Figure copy (BIT) (star, cube, flower, triangles) [1,2]	x	x		✓			x			x	✓		
Design copy test (house, flower, clock, diamond) [116]	x	x		✓			x			X	✓		
Copy a daisy/Daisy drawing [103]	x	x		✓			x			X	✓		
Two daisy figure /Complex flower copying task [117]	x	x		✓			x			x	✓		
Rey-Osterrieth complex figure test, copy [118]	x	x		✓			x			x	✓		
**Drawing tasks**													
Clock drawing task (19 pt scale) [119]	x	x		✓		x	x			x	✓		
Clock drawing test (Freedman 15 pt scale) [120]	x	x		✓		x	x			x	✓		
Clock drawing task (CLOX) (15pts) [121]	x	x		✓		x	x			x	✓		
Clock drawing (from memory) (12 pt scale) [103,122]	x	x		✓		x	x			x	✓		
Clock drawing test (Sunderland/Manos 10 pts) [123,124]	x	x		✓		x	x			x	✓		
Clock drawing test (7 pt scale) - Watson system [5]; Seven Minute Screen (Solomon & Pendlebury) [124]	x	x		✓		x	x			x	✓		
Clock drawing test (4 pt scale) (printed 8cm circle) [125]	x	x		✓		x	x			x	✓		
Clock drawing (4 pt scale) [126]	x	x		✓		x	x			x	✓		
OT-APST (Occupational Therapy Adult Perceptual Screening Test) - clock drawing subtest [22]	x	x		✓		x	x			x	✓		
Clock drawing test (2 pt scale) [14,127]	x	x		✓		x	x			x	✓		
Clock drawing task (Copy) (impaired/normal) [81]	x	x		✓		x	x			x	✓		
Number clockface (printed circle) (impaired/normal) [16]	x	x		✓		x	x			x	✓		
Five-Point test (Regard 1982) [128] (5 Dot test)	✓			✓			x			x	✓		
BIT representational drawing (clock, person, butterfly) [1,2]	x	x		✓		x	x			x	✓		
Draw-A-Man test [129]	x	x		✓		x	x			x	✓		
Writing task [14]	✓			✓		x	x			x	✓	✓	
Arrow location in 3 conditions (blank, lines, numbers) [130]	✓			✓			x			x	✓		

✓ Tool differentiates the neglect subtype.
x Deficient performance may be attributable to the subtype, but the scoring does not allow for it to be differentiated from other subtypes.
? Unable to determine from documented protocols.
(x) Alternative protocols of the test involve this subtype (for example motor response if unable to verbalise response)

Tool name (reference)	Egocentric	Allocentric	Personal	Peripersonal	Extrapersonal	Representation	Visual	Tactile	Auditory	Motor	Impairment	Activity Limitation	Participation Restriction
**Reading tasks**													
Sentence reading [131]	✓	x		✓			✓				✓	✓	
Reading task/ text reading [14]	✓	x		✓			✓				✓	✓	
Sentence reading [123,132]	✓	x		✓			✓				✓	✓	
Single word reading (Vallar 1996) [123]	x	x		✓			✓				✓		
Text reading [133]	✓	x		✓			✓				✓	✓	
Indented paragraph reading test [134]	✓	x		✓			✓				✓	✓	
Indented paragraph test modified [46,134,135]	✓			✓			✓				✓	✓	
Reading of compound-words [133]	✓	✓		✓			✓				✓		
Reading task (individual words & sentences) [35]	✓	✓		✓			✓				✓	✓	
Reading task [136]	✓	x		✓			✓				✓	✓	
Munich reading tests [137]	✓			✓			✓				✓	✓	
Menu subtests of the BIT [1]	x	x		✓			✓				✓	✓	
Article reading subtests of the BIT [1]	x	x		✓			✓				✓	✓	
Neglect dyslexia test - Two-word phrases [138]	x	x		✓			✓				✓		
Neglect dyslexia test - Single words [138]	x	x		✓			✓				✓		
Word reading test (WR; range 0–35) [139]	x	x		✓			✓				✓	✓	
Experimental word reading test (EWR; range:0–35) [139]	x	x		✓			✓				✓		
Single word reading task [140]	x	x		✓			✓				✓		
Article reading (Finnish folk story) [89]	✓	✓		✓			✓				✓	✓	
Neglect dyslexia test (Korean) [141]	x	x		✓			✓				✓		
Conventional text reading task [140]	x	x		✓			✓				✓	✓	
Number reading task (Max 9pts) [121]	x	x		✓			✓				✓		
**Other visual tasks**													
Ravens Coloured Progressive Matrices (RCPM) (proportion of right and left responses) [46]	✓			✓			✓			(x)	✓		
Visual-counting test [74]	✓			✓			✓				✓		
Picture scanning subtest of BIT [1]	✓			✓			✓				✓		
Overlapping figures test [14,142]	✓	✓		✓			✓				✓		

✓ Tool differentiates the neglect subtype.
x Deficient performance may be attributable to the subtype, but the scoring does not allow for it to be differentiated from other subtypes.
? Unable to determine from documented protocols.
(x) Alternative protocols of the test involve this subtype (for example motor response if unable to verbalise response)

Tool name (reference)	Egocentric	Allocentric	Personal	Peripersonal	Extrapersonal	Representation	Visual	Tactile	Auditory	Motor	Impairment	Activity Limitation	Participation Restriction
**Other visual tasks (continued)**													
Object search task [143]	x	x		✓			✓			(x)	✓		
Independent segment estimation task [71]	x	x		✓		x	x				✓		
Chimeric object naming test [144,145]	x	x		✓			✓				✓		
Chimeric figures [146]	x	x		✓			✓				✓		
Chimeric faces [147]	x	x		✓			✓				✓		
Wundt–Jastrow area illusion test [148]	x	x		✓			✓				✓		
Eye tracking free exploration task [121]	✓			✓			✓				✓		
Eye tracking visual search task [121]	✓			✓			✓				✓		
Free visual exploration task [149]	✓			✓			✓				✓		
Gaze and head orientation at rest and during active visual exploration [150]	✓			✓			✓			x	✓		
Spontaneous head and eye gaze [151]	✓				✓		✓			x	✓		
Video-oculography with FVE paradigm [152]	✓			✓			✓				✓		
Eye tracking with IADL task [153]	✓			x	x		✓				✓	✓	
Inhibition of return (IOR) - covert/overt attention [154]	✓			✓			✓				✓		
Orientation singletons [155]	✓			✓			✓				✓		
**Representational tasks**													
The “Bisiach-Ogden test” [156]	x	x		✓		x	x				✓		
Familiar Square Description Test [157,158]	✓					✓					✓		
3 Roman Squares [159]	✓					✓					✓		
Imaginal RT task (towns in France) [160]	✓					✓				x	✓		
Towns on coast of Italy [159]	✓					✓					✓		
Countries of Europe [159]	✓					✓					✓		
Description from memory of map of France [161]	✓					✓					✓		
map of France (and inverted map) [162]	✓					✓					✓		
O’Clock Test [158,163]	x	x		✓		✓					✓		
Map of France (eyes open and eyes closed) [161]	✓					✓					✓		
Tactuo-Motor search (TMS) task [89]	x	x		✓		x	x				✓		

✓ Tool differentiates the neglect subtype.
x Deficient performance may be attributable to the subtype, but the scoring does not allow for it to be differentiated from other subtypes.
? Unable to determine from documented protocols.
(x) Alternative protocols of the test involve this subtype (for example motor response if unable to verbalise response)

Tool name (reference)	Egocentric	Allocentric	Personal	Peripersonal	Extrapersonal	Representation	Visual	Tactile	Auditory	Motor	Impairment	Activity Limitation	Participation Restriction
**Personal space tasks**													
Fluff test [164]	✓		✓			x		x		x	✓		
modified version of the Fluff Test (6x Left, 4 Right) [28]	✓		✓			x		x		x	✓		
modified version of Fluff test - (12 x upper body) [165]	✓		✓			x		x		x	✓		
modified version of Fluff test - (5xL side, 2xR) [126]	✓		✓			x		x		x	✓		
Vest Test [166]	✓		✓			x		x		x	✓		
Personal neglect evaluation [89]	✓		✓			x	x			x	✓		
Extension of Personal neglect test [167]	✓		✓			x	x			x	✓		
Personal neglect test /Bisiach test (Touching hand) [168]	✓		✓			x	x			x	✓		
**Far space tasks**													
Dublin Extrapersonal neglect assessment (DENA) [169]	✓				✓		✓			x	✓	✓	
Room description task [4]	✓				✓		✓				✓		
The Visual Scanning Test (VST) [170]	✓				✓		✓				✓		
Large screen tasks (Active space): Detection task and crash task (Single and dual task) [171]	✓				✓		✓			?	✓		
Slide (far space) test [89]	✓				✓		✓				✓		
laser line-bisection task [28]	x	x			✓		x			x	✓		
**Far and near space tasks**													
Line bisection and Bells Test in near and far space [172]	✓			✓	✓		x			x	✓		
Digitized object cancellation in peripersonal and extrapersonal space) [173]	✓			✓	✓		x			x	✓		
Digitized line bisection task in peripersonal and extrapersonal space) (3 x 22cm lines on page x 4) [173]	✓			✓	✓		x			x	✓		
Line bisection task (10 x lines at 50cm, 1.5m, 3m) [174]	✓			✓	✓		x			x	✓		
Letter cancellation task [173]	✓			✓	✓		x			x	✓		
Schenkenberg line bisection & star cancellation test in near and far extrapersonal space [174,175]	✓			✓	✓		x			x	✓		
Wundt-Jastro area illusion: peri-personal and extra-personal presentation [176]	x	x		✓	✓		✓				✓		
Doorway bisection task [174]	✓			✓	✓		x			x	✓	✓	

✓ Tool differentiates the neglect subtype.
x Deficient performance may be attributable to the subtype, but the scoring does not allow for it to be differentiated from other subtypes.
? Unable to determine from documented protocols.
(x) Alternative protocols of the test involve this subtype (for example motor response if unable to verbalise response)

Tool name (reference)	Egocentric	Allocentric	Personal	Peripersonal	Extrapersonal	Representation	Visual	Tactile	Auditory	Motor	Impairment	Activity Limitation	Participation Restriction
**Personal, far and near space tasks**													
Halifax Visual Scanning Test (HVST)[177]	✓		✓	✓	✓		✓				✓		
**Dynamic tasks – VR and computer-based**													
Test Battery for Attentional Performance (TAP) - neglect subtest [178]	✓			✓			✓			?	✓		
Virtual Reality Lateralized Attention Test (VRLAT) [28, 179]	✓			x	x		x			x	✓	✓	
Virtual reality wheelchair navigation test [180]	✓			x	x		x			x	✓	✓	
VR-DiSTRO (VR-SCT, VR-LB, VR-BTT, VR-EXT)[181]	✓			✓			x			x	✓		
Virtual Environment for Spatial Neglect Assessment (VESNA) [182]	✓			x	x		x			x	✓		
Virtual reality assessment for visuospatial neglect [183]	✓			?	?		✓			x	✓	✓	
VR Assessment and Training system for Unilateral Neglect[184]	✓			?	?		✓			x	✓		
VR -three-dimensional virtual street crossing program[185,186]	✓			x	x		✓				✓	✓	
Virtual Street Crossing system [187]	✓			x	x		x			x	✓	✓	
Ecological VR-based Evaluation of Neglect Symptoms (EVENS) [188]	✓			x	x		x			x	✓	✓	
VR-based goal-directed locomotion task [175]	✓				✓	x	x			x	✓	✓	
Visual Search Task - Cognitive rehabilitation platform Guttmann-NeuroPersonal Trainer (García-Molina et al., 2010; Solana et al., 2014; Solana et al., 2015).	✓			✓			x			x	✓		
VISSTA (Visual Spatial Search Task) [189]	✓			?			✓				✓		
Computerised Table Setting Test [94]	✓			✓			x			x	✓	✓	
MonAmour Test (Robot Diagnosis Test for Hemineglect) [178]	✓	✓		✓			✓				✓		
Starry Night Test (SNT) [190]	✓			✓			✓				✓		
Adaptive visual conjunction search task [191,192]	✓			?	?		✓				✓		
Vienna Test System - peripheral perception subtest [193]	✓			✓			✓			x	✓		
TVA - whole or partial report task [194]	✓			✓			✓				✓		
Useful Field of View (UFOV) [195]	✓			✓			x			x	✓		
Simulated driving task [196,197]	✓				✓		x			x	✓	✓	

✓ Tool differentiates the neglect subtype.
x Deficient performance may be attributable to the subtype, but the scoring does not allow for it to be differentiated from other subtypes.
? Unable to determine from documented protocols.
(x) Alternative protocols of the test involve this subtype (for example motor response if unable to verbalise response)

Tool name (reference)	Egocentric	Allocentric	Personal	Peripersonal	Extrapersonal	Representation	Visual	Tactile	Auditory	Motor	Impairment	Activity Limitation	Participation Restriction
**Dual tasks**													
E-prime single task & dual task [198,199]	✓			✓			✓				✓		
Computerized Visual RT Task: single (CVRT) and dual (CVRT-D) task [196]	✓			✓			✓				✓		
Multi-tasking paradigm (Single and Dual Tasks) [200]	✓			✓			✓		✓		✓		
Dual task test [180]	✓			✓			✓				✓		
**Reaction time tasks**													
Posner cuing task - Reaction time test / Valid versus invalid cue paradigm [120]	✓			✓			✓				✓		
Spatial cueing (Posner) task [201]	✓			✓			✓				✓		
Eye movement RT task [202]	✓			✓			✓				✓		
Spatial orientation task [203]	✓			✓			✓				✓		
Perceptual RT task/ Reaction time task [69,160]	✓			✓			✓				✓		
**Auditory tasks**													
Dichotic listening task [204]	✓								✓		✓		
Temporal Order Judgement (TOJ) task [205]	✓			✓			✓			(x)	✓		
Lateralisation of dichotic sound [206]	✓								✓		✓		
Auditory Spatial Discrimination Task [207]	✓								✓		✓		
Psychoacoustic pattern discrimination test (PPDT) modified non-adaptive version [208]	✓								✓		✓		
Visual and Auditory TOJ Task [209]	✓			✓			✓		✓		✓		
**Extinction Tasks**													
National Institute of Health Stroke Scale (NIHSS) - Neglect /extinction item	x		x	x			x	x	x		✓		
Cog-4 (NIHSS Subscale of 4 Cognitive Items) [210]	x		x	x			x	x	x		✓		
Tactile extinction test DSS (8L, 8R, 8B) [33]	x		x					x			✓		
Visual extinction test DSS (8, 8, 8) [33]	x		x				x				✓		
Thermal pain DSS protocol [33]	x		x					x			✓		
DSS with electromagnetic solenoids (18x/12x + 6 catch trials) [211]	x		x					x			✓		
Confrontation method of extinction (Tactile, auditory, visual) (score 0-9) [212]	x		x	x	x		x	x	x		✓		
Computerised test of visual neglect and extinction [213]	x			x			x			x	✓		
Novel computerised test for visual extinction/neglect [214]	x			?			x				✓		

✓ Tool differentiates the neglect subtype.
x Deficient performance may be attributable to the subtype, but the scoring does not allow for it to be differentiated from other subtypes.
? Unable to determine from documented protocols.
(x) Alternative protocols of the test involve this subtype (for example motor response if unable to verbalise response)

Tool name (reference)	Egocentric	Allocentric	Personal	Peripersonal	Extrapersonal	Representation	Visual	Tactile	Auditory	Motor	Impairment	Activity Limitation	Participation Restriction
**ADL based tasks**													
Catherine Bergego Scale (CBS) [215]	✓		x	x	x	x	x	x	x	x		✓	
Catherine Bergego Scale via KF-NAP [216,217]	✓		x	x	x	x	x	x	x	x		✓	
Subjective Neglect Questionnaire [218]	✓	x	x	x	x	x	x	x	x	x		✓	
ADL checklist [8]	✓		x	x	x		x	x	x	x		✓	✓
Árnadóttir OT-ADL Neurobehavioral Evaluation (A-ONE) [219]	✓		x	x		x	x		x	x		✓	
Catherine Bergego Scale Dutch version [220,221]	✓		x	x	x	x	x	x	x	x		✓	
Catherine Bergego Scale -extrapersonal items (CBS-E) [169]	✓				✓		x		x	x		✓	
OT checklist of everyday neglect behaviours [2]	✓		x	x	x		x	x	x	x		✓	✓
Modified checklist of everyday neglect behaviors [2,6]	✓	x	x	x	x	x	x	x	x	x		✓	✓
Novel ADL-based neglect test (Activities of Daily Living Scale) [11]	✓	x	x	x	x	x	x			x		✓	
Semi-structured scale for the functional evaluation of extrapersonal neglect [222]	✓			✓	✓		✓			x		✓	
Semi-structured scale for the functional evaluation of personal neglect [223]	✓		✓			x	x			x		✓	
Tea Making (TM) Task [224]	✓			✓			x			x		✓	
Document Filing (DF) Task [224]	✓			✓			x			x		✓	
Comb and razor test [225]	✓		✓			x	x			x		✓	
Reformulated comb and razor test [225,226]	✓		x			x	x			x		✓	
Key removal, grocery naming, face washing, tray wiping [165]	✓		x	x			x			x		✓	
Visual Neglect Recovery Index (VNRI) [227]	✓			x	x		x			x	✓	✓	
Naturalistic Action Test (NAT) [228] (Lateralised Attention Score) [229]	✓			x	x		x			x	✓	✓	
Multi-Level Action Test (MLAT) (making toast, wrapping present, packing lunchbox) [230]	✓			✓			x			x		✓	

✓ Tool differentiates the neglect subtype.
x Deficient performance may be attributable to the subtype, but the scoring does not allow for it to be differentiated from other subtypes.
? Unable to determine from documented protocols.
(x) Alternative protocols of the test involve this subtype (for example motor response if unable to verbalise response)

Tool name (reference)	Egocentric	Allocentric	Personal	Peripersonal	Extrapersonal	Representation	Visual	Tactile	Auditory	Motor	Impairment	Activity Limitation	Participation Restriction
**Mobility based tasks**													
Mobility Assessment Course [85,231]	✓			x	x		✓			x		✓	
Doorway Accuracy Test (DAT) [232]	✓			x	x		x			x		✓	
Wheelchair assessment Course (WAC) [233]	✓			x	x		x			x		✓	
Cone Evasion Walk Test [234]	✓			x	x		✓			x		✓	
Moss–Magee Wheelchair Navigation Test (MMWNT) [45,180,235]	✓			x	x		x			x	✓	✓	
modified MossReal World Navigation (RWN) test [28, 180]	✓			x	x		x			x	✓	✓	
Wheelchair obstacle course [177,236]	✓			x	x		x			x	✓	✓	
Wheelchair Collision Test (WCT) [237]	✓			x	x		x			x	✓	✓	
Wheelchair assessment Course (WAC) [233]	✓			x	x		x			x	✓	✓	

✓ Tool differentiates behaviours for the neglect subtype.
x Deficient performance may be attributable to the subtype, but the soring does not allow for it to be differentiated from other subtypes.
? Unable to determine from documented protocols.
(x) Alternative protocols of the test involve this subtype (for example motor response if unable to verbalise response)

References:

[1] Wilson B, Cockburn J, Halligan P. Development of a behavioral test of visuospatial neglect. Arch Phys Med Rehabil 1987;68:98–102.
[2] Halligan PW, Cockburn J, Wilson BA. The behavioural assessment of visual neglect. Neuropsychological Rehabilitation 1991;1:5–32. https://doi.org/10.1080/09602019108401377.
[3] Appelros P, Karlsson G, Thorwalls A, Tham K, Nydevik I. Unilateral neglect: further validation of the baking tray task. Journal of Rehabilitation Medicine 2004;36:258-61.
[4] Stone SP, Wilson B, Wroot A, Halligan PW, Lange LS, Marshall JC, et al. The assessment of visuo-spatial neglect after acute stroke. J Neurol Neurosurg Psychiatry 1991;54:345–50.
[5] Fong KNK, Chan MKL, Chan BYB, Ng PPK, Fung ML, Tsang MHM, et al. Reliability and validity of the Chinese behavioural inattention test-Hong Kong version (CBIT-HK) for patients with stroke and unilateral neglect. Hong Kong Journal of Occupational Therapy 2007;17:23–33. https://doi.org/10.1016/S1569-1861%2807%2970004-9.
[6] Tanaka T, Sugihara S, Nara H, Ino S, Ifukube T. A preliminary study of clinical assessment of left unilateral spatial neglect using a head mounted display system (HMD) in rehabilitation engineering technology. Journal of NeuroEngineering and Rehabilitation 2005;2:31. https://doi.org/10.1186/1743-0003-2-31.
[7] Isiai S. Behavioural Inattention Test, Japanese edition. Tokyo, Tapan: Shinkoh Igaku Shuppan, Co Ltd; 1999.
[8] Hartman-Maeir A, Katz N. Validity of the Behavioral Inattention Test (BIT): Relationships with functional tasks. American Journal of Occupational Therapy 1995;49:507–16. https://doi.org/10.5014/ajot.49.6.507.
[9] Katz N, Averbuch S, Itzkovich M. Hebrew translation of behavioural inattention test manual. Fareham, Hants, England: Thames Valley Test Co; 1991.
[10] Ulm L, Wohlrapp D, Meinzer M, Steinicke R, Schatz A, Denzler P, et al. A circle-monitor for computerised assessment of visual neglect in peripersonal space. PloS One 2013;8:e82892. https://doi.org/10.1371/journal.pone.0082892.
[11] Eschenbeck P, Vossel S, Weiss PH, Saliger J, Karbe H, Fink GR. Testing for neglect in right-hemispheric stroke patients using a new assessment battery based upon standardized activities of daily living (ADL). Neuropsychologia 2010;48:3488–96. https://doi.org/10.1016/j.neuropsychologia.2010.07.034.
[12] Sánchez-Cabeza A, Huertas-Hoyas E, Máximo-Bocanegra N, Martínez-Piédrola RM, Pérez-de-Heredia-Torres M, Alegre-Ayala J. Spanish transcultural adaptation and validity of the behavioral inattention test. Occup Ther Int 2017;2017. https://doi.org/10.1155/2017/1423647.
[13] Leibovitch FS, Vasquez BP, Ebert PL, Beresford KL, Black SE. A short bedside battery for visuoconstructive hemispatial neglect: Sunnybrook Neglect Assessment Procedure (SNAP). Journal of Clinical and Experimental Neuropsychology 2012;34:359–68. https://doi.org/10.1080/13803395.2011.645016.

[14] Azouvi P, Samuel C, Louis-Dreyfus A, Bernati T, Bartolomeo P, Beis JM, et al. Sensitivity of clinical and behavioural tests of spatial neglect after right hemisphere stroke. Journal of Neurology Neurosurgery and Psychiatry 2002;73:160–6. https://doi.org/10.1136/jnnp.73.2.160.

[15] Azouvi P, Bartolomeo P, Beis J-M, Perennou D, Pradat-Diehl P, Rousseaux M. A battery of tests for the quantitative assessment of unilateral neglect. Restorative Neurology and Neuroscience 2006;24:273–85.

[16] Friedman PJ, Leong L. The Rivermead Perceptual Assessment Battery in acute stroke. The British Journal of Occupational Therapy 1992;55:233–7. https://doi.org/10.1177/030802269205500608.

[17] Williams PM, Johnson C, Swan S, Barber C, Murphy P, Devine J, et al. The northwick park examination of cognition: A brief cognitive assessment tool for use in acute stroke services. International Journal of Therapy and Rehabilitation 2016;23:314–22.

[18] Quinn TJ, Livingstone I, Weir A, Shaw R, Breckenridge A, McAlpine C, et al. Accuracy and Feasibility of an Android-Based Digital Assessment Tool for Post Stroke Visual Disorders-The StrokeVision App. Front Neurol 2018;9:146. https://doi.org/10.3389/fneur.2018.00146.

[19] Rowe F.J., Hepworth L.R., Hanna K.L., Howard C. Visual Impairment Screening Assessment (VISA) tool: Pilot validation. BMJ Open 2018;8:e020562. https://doi.org/10.1136/bmjopen-2017-020562.

[20] Sodring KM, Laake K, Sveen U, Wyller TB, Bautz-Holter E. Validation of the screening instrument for neuropsychological impairment in stroke. Physiother Res Int 1998;3:15–26.

[21] Pizzamiglio L, Judica A, Razzano C, Zoccolotti P. Toward a comprehensive diagnosis of visual-spatial disorders in unilateral brain damaged patients. Evaluación Psicológica 1989;5:199–218.

[22] Cooke D.M., McKenna K., Fleming J., Darnell R. The reliability of the Occupational Therapy Adult Perceptual Screening Test (OT-APST). Br J Occup Ther 2005;68:509–17.

[23] Boys M, Fisher P, Holzberg C, Reid DW. The OSOT Perceptual Evaluation: a research perspective. The American Journal of Occupational Therapy 1988:92–8.

[24] Desrosiers J, Mercier L, Rochette A. Fidélité test-retest et interjuges de la version française du OSOT Perceptual Evaluation. Can J Occup Ther 1999;66:134–9. https://doi.org/10.1177/000841749906600305.

[25] Vaes N, Lafosse C, Nys G, Schevernels H, Dereymaeker L, Oostra K, et al. Capturing peripersonal spatial neglect: An electronic method to quantify visuospatial processes. Behavior Research Methods 2015;47:27–44. https://doi.org/10.3758/s13428-014-0448-0.

[26] Gauthier L, Dehaut F, Joanette Y. The bells test: a quantitative and qualitative test for visual neglect. International Journal of Clinical Neuropsychology 1989;11:49–54.

[27] Basagni B, De Tanti A, Damora A, Abbruzzese L, Varalta V, Antonucci G, et al. The assessment of hemineglect syndrome with cancellation tasks: a comparison between the Bells test and the Apples test. Neurol Sci 2017;38:2171–6. https://doi.org/10.1007/s10072-017-3139-7.

[28] Buxbaum LJ, Dawson AM, Linsley D. Reliability and validity of the Virtual Reality Lateralized Attention Test in assessing hemispatial neglect in right-hemisphere stroke. Neuropsychology 2012;26:430–41. https://doi.org/10.1037/a0028674.

[29] Friedman PJ. The Star Cancellation Test in acute stroke. Clinical Rehabilitation 1992;6:23–30. https://doi.org/10.1177/026921559200600104.

[30] Albert ML. A simple test of visual neglect. Neurology 1973;23:658–64. https://doi.org/10.1212/WNL.23.6.658.

[31] Fullerton KJ, Mcsherry D, Stout RW. Albert's test: A neglected test of perceptual neglect. The Lancet 1986;327:430–2. https://doi.org/10.1016/S0140-6736(86)92381-0.

[32] Weintraub S, Mesulam MM. Principles of behavioral neurology. Mental state assessment of young and elderly adults in behavioral neurology, Philadelphia: PA: Davis FA; 1985, p. 71–123.

[33] Liu CC, Veldhuijzen DS, Ohara S, Winberry J, Greenspan JD, Lenz FA. Spatial attention to thermal pain stimuli in subjects with visual spatial hemi-neglect: Extinction, mislocalization and misidentification of stimulus modality. Pain 2011;152:498–506. https://doi.org/10.1016/j.pain.2010.10.017.

[34] Toba MN, Rabuffetti M, Duret C, Pradat-Diehl P, Gainotti G, Bartolomeo P. Component deficits of visual neglect: "Magnetic" attraction of attention vs. impaired spatial working memory. Neuropsychologia 2018;109:52–62. https://doi.org/10.1016/j.neuropsychologia.2017.11.034.

[35] Khurshid S, Trupe LA, Newhart M, Davis C, Molitoris JJ, Medina J, et al. Reperfusion of specific cortical areas is associated with improvement in distinct forms of hemispatial neglect. Cortex 2012;48:530–9. https://doi.org/10.1016/j.cortex.2011.01.003.

[36] Pia L, Ricci R, Gindri P, Vallar G. Drawing perseveration in neglect: Effects of target density. Journal of Neuropsychology 2013;7:45–57. https://doi.org/10.1111/j.1748-6653.2012.02034.x.

[37] Halligan P, Wilson B, Cockburn J. A short screening test for visual neglect in stroke patients. Int Disabil Stud 1990;12:95–9.

[38] Wansard M, Meulemans T, Gillet S, Segovia F, Bastin C, Toba MN, et al. Visual neglect: Is there a relationship between impaired spatial working memory and re-cancellation? Experimental Brain Research 2014;232:3333–43. https://doi.org/10.1007/s00221-014-4028-4.

[39] Edgeworth Jennifer A, MacMillan TM, Robertson IH. The balloons test manual 1998.

[40] Spreij LA, Ten Brink AF, Visser-Meily JMA, Nijboer TCW. Simulated driving: The added value of dynamic testing in the assessment of visuo-spatial neglect after stroke. Journal of Neuropsychology 2020;14:28–45. https://doi.org/10.1111/jnp.12172.

[41] Broeren J, Samuelsson H, Stibrant-Sunnerhagen K, Blomstrand C, Rydmark M. Neglect assessment as an application of virtual reality. Acta Neurologica Scandinavica 2007;116:157–63. https://doi.org/10.1111/j.1600-0404.2007.00821.x.

[42] Rabuffetti M, Ferrarin M, Spadone R, Pellegatta D, Gentileschi V, Vallar G, et al. Touch-screen system for assessing visuo-motor exploratory skills in neuropsychological disorders of spatial cognition. Medical and Biological Engineering and Computing 2002;40:675–86.

[43] Vallar G, Guariglia C, Nico D, Bisiach E. Spatial hemineglect in back space. Brain 1995;118:467–72. https://doi.org/10.1093/brain/118.2.467.

[44] Diller L, Weinberg J. Hemi-inattention in rehabilitation. The evolution of a rational remediation program. In: Weinstein EA, Friedland RP, editors. Hemi-inattention and hemisphere specialization: Advances in neurology, New York: Raven Press; 1977, p. 62–82.

[45] Webster JS, Rapport LJ, Godlewski MC, Abadee PS. Effect of attentional bias to right space on wheelchair mobility. Journal of Clinical and Experimental Neuropsychology 1994;16:129–37. https://doi.org/10.1080/01688639408402623.

[46] Bachman L, Fein G, Davenport L, Price L. The indented paragraph reading test in the assessment of left hemi- neglect. Archives of Clinical Neuropsychology 1993;8:485–96. https://doi.org/10.1016/0887-6177%2893%2990049-7.

[47] Verhoeven CL, Post MW, Schiemanck SK, van Zandvoort MJ, Vrancken PH, van Heugten CM. Is Cognitive Functioning 1 Year Poststroke Related to Quality of Life Domain? Journal of Stroke & Cerebrovascular Diseases 2011;20:450-458 9p.

[48] Donnelly N, Guest R, Fairhurst M, Potter J, Deighton A, Patel M. Developing algorithms to enhance the sensitivity of cancellation tests of visuospatial neglect. Behavior Research Methods, Instruments, & Computers : A Journal of the Psychonomic Society, Inc 1999;31:668–73.

[49] Sea M-JC, Henderson A. The reliability and validity of visuospatial inattention tests with stroke patients. Occupational Therapy International 1994;1:36–48. https://doi.org/10.1002/oti.6150010106.

[50] Chen Sea M, Cermack SA, Henderson A. Performance of normal Chinese adults and right CVA patients on the Random Chinese Word Cancellation Test. Clinical Neuropsychologist 1993;7:239–49. https://doi.org/10.1080/13854049308401896.

[51] Chen-Sea M. Unilateral neglect and functional significance among patients with stroke. Occupational Therapy Journal of Research 2001;21:223-240 18p.

[52] Goldenberg G, Münsinger U, Karnath HO. Severity of neglect predicts accuracy of imitation in patients with right hemisphere lesions. Neuropsychologia 2009;47:2948–52. https://doi.org/10.1016/j.neuropsychologia.2009.06.024.

[53] Wojciulik E, Rorden C, Clarke K, Husain M, Driver J. Group study of an "undercover" test for visuospatial neglect: Invisible cancellation can reveal more neglect than standard cancellation. Journal of Neurology, Neurosurgery and Psychiatry 2004;75:1356–8. https://doi.org/10.1136/jnnp.2003.021931.

[54] Nijboer TCW, Kollen BJ, Kwakkel G. Time course of visuospatial neglect early after stroke: A longitudinal cohort study. Cortex 2013;49:2021–7. https://doi.org/10.1016/j.cortex.2012.11.006.

[55] Bickerton WL, Samson D, Williamson J, Humphreys GW. Separating forms of neglect using the Apples Test: Validation and functional prediction in chronic and acute stroke. Neuropsychology 2011;25:567–80. https://doi.org/10.1037/a0023501.

[56] Bickerton W-L, Demeyere N, Francis D, Kumar V, Remoundou M, Balani A, et al. The BCoS cognitive profile screen: Utility and predictive value for stroke. Neuropsychology 2015;29:638–48. https://doi.org/10.1037/neu0000160.

[57] Kong A.P.-H., Chan J., Lau J.K.-L., Bickerton W.-L., Weekes B., Humphreys G. Developing a Cantonese Version of Birmingham Cognitive Screen for Stroke Survivors in Hong Kong. Commun Disord Q 2018;39:387–401. https://doi.org/10.1177/1525740117720382.

[58] Pan X, Chen H, Bickerton WL, Lau JKL, Kong APH, Rotshtein P, et al. Preliminary findings on the reliability and validity of the cantonese birmingham cognitive screen in patients with acute ischemic stroke. Neuropsychiatric Disease and Treatment 2015;11:2377–90. https://doi.org/10.2147/NDT.S85698.

[59] Demeyere N, Riddoch MJ, Slavkova ED, Bickerton WL, Humphreys GW. The Oxford Cognitive Screen (OCS): validation of a stroke-specific short cognitive screening tool. Psychol Assess 2015;27:883–94. https://doi.org/10.1037/pas0000082.

[60] Kong A.P.-H., Lam P.H.-P., Ho D.W.-L., Lau J.K., Humphreys G.W., Riddoch J., et al. The Hong Kong version of the Oxford Cognitive Screen (HK-OCS): validation study for Cantonese-speaking chronic stroke survivors. Aging Neuropsychol Cogn 2016;23:530–48. https://doi.org/10.1080/13825585.2015.1127321.

[61] Shendyapina M, Kuzmina E, Kazymaev S, Petrova A, Demeyere N, Weekes BS. The Russian version of the Oxford Cognitive Screen: Validation study on stroke survivors. Neuropsychology 2019;33:77–92. https://doi.org/10.1037/neu0000491.

[62] Chen P, Caulfield MD, Hartman AJ, O'Rourke J, Toglia J. Assessing viewer-centered and stimulus-centered spatial bias: The 3s spreadsheet test version 1. Appl Neuropsychol Adult 2017;24:532–9. https://doi.org/10.1080/23279095.2016.1220382.

[63] Ota H, Fujii T, Suzuki K, Fukatsu R, Yamadori A. Dissociation of body-centered and stimulus-centered representations in unilateral neglect. Neurology 2001;57:2064–9.

[64] Marsh EB, Hillis AE. Dissociation between egocentric and allocentric visuospatial and tactile neglect in acute stroke. Cortex 2008;44:1215–20. https://doi.org/10.1016/j.cortex.2006.02.002.

[65] Mancuso M, Rosadoni S, Capitani D, Bickerton WL, Humphreys GW, De Tanti A, et al. Italian standardization of the Apples Cancellation Test. Neurological Sciences 2015;36:1233–40. https://doi.org/10.1007/s10072-015-2088-2.

[66] Goedert KM, Chen P, Botticello A, Masmela JR, Adler U, Barrett AM. Psychometric evaluation of neglect assessment reveals motor-exploratory predictor of functional disability in acute-stage spatial neglect. Archives of Physical Medicine and Rehabilitation 2012;93:137–42. https://doi.org/10.1016/j.apmr.2011.06.036.

[67] Na DL, Adair JC, Williamson DJG, Schwartz RL, Haws B, Heilman KM. Dissociation of sensory-attentional from motor-intentional neglect. Journal of Neurology Neurosurgery and Psychiatry 1998;64:331–8.

[68] Husain M, Mattingley JB, Rorden C, Kennard C, Driver J. Distinguishing sensory and motor biases in parietal and frontal neglect. Brain 2000;123:1643–59.

[69] Bartolomeo P, D'Erme P, Perri R, Gainotti G. Perception and action in hemispatial neglect. Neuropsychologia 1998;36:227–37. https://doi.org/10.1016/S0028-3932(97)00104-8.

[70] Bisiach E, Ricci R, Lualdi M, Colombo MR. Perceptual and response bias in unilateral neglect: two modified versions of the Milner Landmark Task. Brain & Cognition 1998;37:369-386

[71] Riestra AR, Crucian GP, Burks DW, Womack KB, Heilman KM. Extinction, working memory, and line bisection in spatial neglect. Neurology 2001;57:147–9. https://doi.org/10.1212/WNL.57.1.147.

[72] Bisiach E, Geminiani G, Berti A, Rusconi ML. Perceptual and premotor factors of unilateral neglect. Neurology 1990;40:1278. https://doi.org/10.1212/WNL.40.8.1278.

[73] Heilman KM, Bowers D, Coslett HB, Whelan H, Watson RT. Directional hypokinesia: Prolonged reaction times for leftward movements in patients with right hemisphere lesions and neglect. Neurology 1985;35:855–9.

[74] Maeshima S, Truman G, Smith DS, Dohi N, Nakai K, Itakura T, et al. Is unilateral spatial neglect a single phenomenon? A comparative study between exploratory-motor and visual-counting tests. J Neurol 1997;244:412–7. https://doi.org/10.1007/s004150050114.

[75] Stone A., Cooke D., Morton D., Steele M. Reliability of revised scoring methods for the Schenkenberg Line Bisection Test with adults following stroke: Preliminary findings. Br J Occup Ther 2019;82:750–8. https://doi.org/10.1177/0308022619866377.

[76] Fortis P, Maravita A, Gallucci M, Ronchi R, Grassi E, Senna I, et al. Rehabilitating patients with left spatial neglect by prism exposure during a visuomotor activity. Neuropsychology 2010;24:681–97. https://doi.org/10.1037/a0019476.

[77] Bailey MJ, Riddoch MJ, Crome P. Evaluation of a test battery for hemineglect in elderly stroke patients for use by therapists in clinical practice. NeuroRehabilitation 2000;14:139–50.

[78] Vallar G, Zilli T, Gandola M, Bottini G. Productive and defective impairments in the neglect syndrome: graphic perseveration, drawing productions and optic prism exposure. Cortex 2006;42:911–20.

[79] Perri R, Bartolomeo P, Gainotti G. Lack of impairments on leftward and rightward line extension tasks in neglect patients. International Journal of Neuroscience 2000;103:101–13.

[80] Lee BH, Kang SJ, Park JM, Son Y, Lee KH, Adair JC, et al. The Character-line Bisection Task: A new test for hemispatial neglect. Neuropsychologia 2004;42:1715–24. https://doi.org/10.1016/j.neuropsychologia.2004.02.015.

[81] Ye L-L, Cao L, Xie H-X, Shan G-X, Zhang Y-M, Song W-Q. Visual-spatial neglect after right-hemisphere stroke: behavioral and electrophysiological evidence. Chin Med J (Engl) 2019;132:1063–70. https://doi.org/10.1097/CM9.0000000000000218.

[82] Karnath HO, Perenin MT. Tactile exploration of peripersonal space in patients with neglect. NeuroReport 1998;9:2273–7. https://doi.org/10.1097/00001756-199807130-00024.

[83] Niemeier M, Karnath HO. Exploratory saccades show no direction-specific deficit in neglect. Neurology 2000;54:515–8.

[84] Machner B, Mah YH, Gorgoraptis N, Husain M. How reliable is repeated testing for hemispatial neglect? Implications for clinical follow-up and treatment trials. Journal of Neurology, Neurosurgery and Psychiatry 2012;83:1032–4. https://doi.org/10.1136/jnnp-2012-303296.

[85] Ten Brink AF, Visser-Meily JM, Nijboer TC. Dynamic assessment of visual neglect: The mobility assessment course as a diagnostic tool. Journal of Clinical and Experimental Neuropsychology 2018;40:161–72. https://doi.org/10.1080/13803395.2017.1324562.

[86] Bisiach E, Ricci R, Silani G, Cossa FM, Crespi MG. Hypermnesia in unilateral neglect. Cortex: A Journal Devoted to the Study of the Nervous System & Behavior 1999;35:701-711

[87] Kleinman JT, Gottesman RF, Davis C, Newhart M, Heidler-Gary J, Hillis AE. Gender differences in unilateral spatial neglect within 24 hours of ischemic stroke. Brain Cogn 2008;68:49–52. https://doi.org/10.1016/j.bandc.2008.02.122.

[88] Butter CM, Mark VW, Heilman KM. An experimental analysis of factors underlying neglect in line bisection. Journal of Neurology 1988;51:1581–3.

[89] Lindell AB, Jalas MJ, Tenovuo O, Brunila T, Voeten MJ, Hamalainen H. Clinical assessment of hemispatial neglect: Evaluation of different measures and dimensions. The Clinical Neuropsychologist 2007;21:479–97. https://doi.org/10.1080/13854040600630061.

[90] Speedie LJ, Wertman E, Verfaellie M, Butter C, Silberman N, Liechtenstein M, et al. Reading direction and spatial neglect. Cortex 2002;38:59–67. https://doi.org/10.1016/S0010-9452(08)70638-5.

[91] Liu M, Chino N, Tuji T, Masakado Y, Hase K, Kimura A. Psychometric properties of the Stroke Impairment Assessment Set (SIAS). Neurorehabilitation & Neural Repair 2002;16:339-351

[92] Tsuji T, Liu M, Sonoda S, Domen K, Chino N. The Stroke Impairment Assessment Set: its internal consistency and predictive validity. Archives of Physical Medicine & Rehabilitation 2000;81:863-868

[93] Heilman KM, Valenstein E. Mechanisms underlying hemispatial neglect. Annals of Neurology 1979;5:166–70. https://doi.org/10.1002/ana.410050210.

[94] Chung SJ, Park E, Ye BS, Lee HS, Chang H-J, Song D, et al. The Computerized Table Setting Test for Detecting Unilateral Neglect. PLoS ONE 2016;11:e0147030. https://doi.org/10.1371/journal.pone.0147030.

[95] Pitzalis S, Spinelli D, Vallar G, Di Russo F. Transcutaneous electrical nerve stimulation effects on neglect: A visual-evoked potential study. Frontiers in Human Neuroscience 2013. https://doi.org/10.3389/fnhum.2013.00111.

[96] Sperber C, Karnath HO. Diagnostic validity of line bisection in the acute phase of stroke. Neuropsychologia 2016;82:200–4. https://doi.org/10.1016/j.neuropsychologia.2016.01.026.

[97] Agrell BM, Dehlin OI, Dahlgren CJ. Neglect in elderly stroke patients: a comparison of five tests. Psychiatry Clin Neurosci 1997;51:295–300.

[98] Bartolomeo P, Chokron S. Egocentric frame of reference: its role in spatial biasafter right hemisphere lesions. Neuropsychologia 1999;37:881–94. https://doi.org/10.1016/S0028-3932(98)00150-X.

[99] Williamson JB, Lamb DG, Burtis DB, Haque S, Zilli EM, Kesayan T, et al. Right hemispatial ipsilesional neglect with chronic right hemisphere strokes. Journal of Clinical and Experimental Neuropsychology 2017:1–10. https://doi.org/10.1080/13803395.2017.1347606.

[100] Wang Q, Sonoda S, Hanamura M, Okazaki H, Saitoh E. Line bisection and rebisection: the crossover effect of space location. Neurorehabilitation & Neural Repair 2005;19:84-92 9p.

[101] Lee BH, Kwon SU, Kwon JC, Baek MJ, Lee KH, Kim GH, et al. Line quadrisection errors in patients with hemispatial neglect. Neurocase 2011;17:372–80. https://doi.org/10.1080/13554794.2010.532136.

[102] Charras P, Lupiáñez J, Bartolomeo P. Assessing the weights of visual neglect: A new approach to dissociate defective symptoms from productive phenomena in length estimation. Neuropsychologia 2010;48:3371–5. https://doi.org/10.1016/j.neuropsychologia.2010.07.006.

[103] Mancini F, Bricolo E, Mattioli FC, Vallar G. Visuo-haptic interactions in unilateral spatial neglect: The cross modal Judd illusion. Frontiers in Psychology 2011;2. https://doi.org/10.3389/fpsyg.2011.00341.

[104] Pirondini E, Goldshuv-Ezra N, Zinger N, Britz J, Soroker N, Deouell LY, et al. Resting-state EEG topographies: Reliable and sensitive signatures of unilateral spatial neglect. NeuroImage-Clin 2020;26:102237. https://doi.org/10.1016/j.nicl.2020.102237.

[105] Anderson B. A mathematical model of line bisection behaviour in neglect. Brain 1996;119 (Pt 3):841–50. https://doi.org/10.1093/brain/119.3.841.

[106] Edwards MG, Humphreys GW. Pointing and grasping in unilateral visual neglect: Effect of on-line visual feedback in grasping. Neuropsychologia 1999;37:959–73. https://doi.org/10.1016/S0028-3932(98)00132-8.

[107] Bailey MJ, Riddoch M, Crome P. Test-retest stability of three tests for unilateral visual neglect in patients with stroke: Star Cancellation, Line Bisection, and the Baking Tray Task. Neuropsychological Rehabilitation 2004;14:403–19. https://doi.org/10.1080/09602010343000282.

[108] Cerrato A, Pacella D, Palumbo F, Beauvais D, Ponticorvo M, Miglino O, et al. E-TAN, a technology-enhanced platform with tangible objects for the assessment of visual neglect: A multiple single-case study. Neuropsychol Rehabil 2020. https://doi.org/10.1080/09602011.2020.1762671.

[109] Wenman R, Bowen A, Tallis RC, Gardener E, Cross S, Niven D. Use of a randomised single case experimental design to evaluate therapy for unilateral neglect. Neuropsychol Rehabil 2003;13:441–59. https://doi.org/10.1080/09602010343000048.

[110] Tyryshkin K, Coderre AM, Glasgow JI, Herter TM, Bagg SD, Dukelow SP, et al. A robotic object hitting task to quantify sensorimotor impairments in participants with stroke. J NeuroEng Rehabil 2014;11:47. https://doi.org/10.1186/1743-0003-11-47.

[111] Brunila T, Jalas M, Lindell JA, Tenovuo O, Hamalainen H. The two part picture in detection of visuospatial neglect. Clin Neuropsychol 2003;17:45–53.

[112] Làdavas E, Umiltà C, Ziani P, Brogi A, Minarini M. The role of right side objects in left side neglect: A dissociation between perceptual and directional motor neglect. Neuropsychologia 1993;31:761–73. https://doi.org/10.1016/0028-3932(93)90127-L.

[113] Gainotti G, Messerli P, Tissot R. Qualitative analysis of unilateral spatial neglect in relation to laterality of cerebral lesions. J Neurol Neurosurg Psychiatry 1972;35:545–50. https://doi.org/10.1136/jnnp.35.4.545.

[114] Johannsen L, Karnath HO. How efficient is a simple copying task to diagnose spatial neglect in its chronic phase? Journal of Clinical and Experimental Neuropsychology 2004;26:251–6. https://doi.org/10.1076/jcen.26.2.251.28085.

[115] Ogden JA. Contralesional neglect of constructed visual images in right and left brain-damaged patients. Neuropsychologia 1985;23:273–7. https://doi.org/10.1016/0028-3932(85)90112-5.

[116] Warren M. Identification of visual scanning deficits in adults after cerebrovascular accident. Journal of Occupational Therapy 1990;44:391–9.

[117] Marshall JC, Halligan PW. Visuo-spatial neglect: A new copying test to assess perceptual parsing. Journal of Neurology 1993;240:37–40. https://doi.org/10.1007/BF00838444.

[118] Osterrieth PA. Le test de copie d'une figure complexe; contribution à l'étude de la perception et de la mémoire. [Test of copying a complex figure; contribution to the study of perception and memory.]. Archives de Psychologie 1944;30:206–356.
[119] Chen P, Goedert KM. Clock Drawing in Spatial Neglect: A Comprehensive Analysis of Clock Perimeter, Placement, and Accuracy. Journal of Neuropsychology May 3;6:270–89. https://doi.org/10.1111/j.1748-6653.2012.02028.x.
[120] Rengachary J, d'Avossa G, Sapir A, Shulman GL, Corbetta M. Is the Posner Reaction Time Test more accurate than clinical tests in detecting left neglect in acute and chronic stroke? Archives of Physical Medicine & Rehabilitation 2009;90:2081-2088 8p. https://doi.org/10.1016/j.apmr.2009.07.014.
[121] Delazer M, Sojer M, Ellmerer P, Boehme C, Benke T. Eye-Tracking Provides a Sensitive Measure of Exploration Deficits After Acute Right MCA Stroke. Front Neurol 2018;9:359. https://doi.org/10.3389/fneur.2018.00359.
[122] Sambo CF, Vallar G, Fortis P, Ronchi R, Posteraro L, Forster B, et al. Visual and spatial modulation of tactile extinction: Behavioural and electrophysiological evidence. Frontiers in Human Neuroscience 2012;6:217. https://doi.org/10.3389/fnhum.2012.00217.
[123] Veronelli L, Vallar G, Marinelli CV, Primativo S, Arduino LS. Line and word bisection in right-brain-damaged patients with left spatial neglect. Experimental Brain Research 2014;232:133–46. https://doi.org/10.1007/s00221-013-3726-7.
[124] Nokleby K, Boland E, Bergersen H, Schanke A-K, Farner L, Wagle J, et al. Screening for cognitive deficits after stroke: A comparison of three screening tools. Clinical Rehabilitation 2008;22:1095–104. https://doi.org/10.1177/0269215508094711.
[125] Ishiai S, Ishiai S, Sugishita M, Sugishita M, Ichikawa T, Ichikawa T, et al. Clock-drawing test and unilateral spatial neglect. Neurology 1993;43:106–10. https://doi.org/10.1212/WNL.43.1_Part_1.106.
[126] Klinke ME, Hjaltason H, Tryggvadóttir GB, Jónsdóttir H. Hemispatial neglect following right hemisphere stroke: clinical course and sensitivity of diagnostic tasks. Top Stroke Rehabil 2018;25:120–30. https://doi.org/10.1080/10749357.2017.1394632.
[127] Rousseaux M, Beis JM, Pradat-Diehl P, Martin Y, Bartolomeo P, Bernati T, et al. A battery for assessing spatial neglect: Norms and effects of age, educational level, sex, hand, and laterality. Revue Neurologique 2001;157:1385–400.
[128] Kaufmann B, Frey J, Pflugshaupt T, Wyss P, Paladini R, Vanbellingen T, et al. The spatial distribution of perseverations in neglect patients during a nonverbal fluency task depends on the integrity of the right putamen. Neuropsychologia 2018;115:42–50. https://doi.org/10.1016/j.neuropsychologia.2018.01.025.
[129] Chen-Sea MJ. Validating the Draw-A-Man Test as a personal neglect test. Journal of Occupational Therapy 2000;54:391–7.
[130] Bartolomeo P, Urbanski M, Chokron S, Chainay H, Moroni C, Siéroff E, et al. Neglected attention in apparent spatial compression. Neuropsychologia 2004;42:49–61. https://doi.org/10.1016/S0028-3932(03)00146-5.
[131] Pizzamiglio L, Antonucci G, Judica A, Montenero P, Razzano C, Zoccolotti P. Cognitive rehabilitation of the hemineglect disorder in chronic patients with unilateral right brain damage. J Clin Exp Neuropsychol 1992;14:901–23.
[132] Zoccolotti P, Antonucci G, Judica A, Montenero P, Pizzamiglio L, Razzano C. Incidence and Evolution of the Hemineglect Disorder in Chronic Patients with Unilateral Right Brain Damage. Null 1989;47:209–16. https://doi.org/10.3109/00207458908987435.
[133] Verdon V, Schwartz S, Lovblad K-O, Hauert C-A, Vuilleumier P. Neuroanatomy of hemispatial neglect and its functional components: A study using voxel-based lesion-symptom mapping. Brain 2010;133:880–94. https://doi.org/10.1093/brain/awp305.
[134] Caplan B. Assessment of unilateral neglect: A new reading test. Journal of Clinical and Experimental Neuropsychology 1987;9:359–64. https://doi.org/10.1080/01688638708405056.
[135] Towle D, Lincoln NB. Use of the Indented Paragraph test with right hemisphere-damaged stroke patients. Br J Clin Psychol 1991;30:37–45.
[136] Emerson RL, García-Molina A, López Carballo J, García Fernández J, Aparicio-López C, Novo J, et al. Visual search in unilateral spatial neglect: The effects of distractors on a dynamic visual search task. Appl Neuropsychol Adult 2019;26:401–10. https://doi.org/10.1080/23279095.2018.1434522.
[137] Cazzoli D., Muri R.M., Schumacher R., Von Arx S., Chaves S., Gutbrod K., et al. Theta burst stimulation reduces disability during the activities of daily living in spatial neglect. Brain 2012;135:3426–39. https://doi.org/10.1093/brain/aws182.
[138] Galletta EE, Campanelli L, Maul KK, Barrett AM. Assessment of neglect dyslexia with functional reading materials. Top 2014;21:75–86. https://doi.org/10.1310/tsr2101-75.
[139] Ronchi R, Algeri L, Chiapella L, Gallucci M, Spada MS, Vallar G. Left neglect dyslexia: Perseveration and reading error types. Neuropsychologia 2016;89:453–64. https://doi.org/10.1016/j.neuropsychologia.2016.07.023.
[140] Reinhart S, Schunck A, Schaadt AK, Adams M, Simon A, Kerkhoff G. Assessing neglect dyslexia with compound words. Neuropsychology 2016;30:869–73. https://doi.org/10.1037/neu0000307.
[141] Lee BH, Suh MK, Kim EJ, Seo SW, Choi KM, Kim GM, et al. Neglect dyslexia: Frequency, association with other hemispatial neglects, and lesion localization. Neuropsychologia 2009;47:704–10. https://doi.org/10.1016/j.neuropsychologia.2008.11.027.

[142] Gainotti G, D'Erme P, Bartolomeo P. Early orientation of attention toward the half space ipsilateral to the lesion in patients with unilateral brain damage. J Neurol Neurosurg Psychiatry 1991;54:1082–9. https://doi.org/10.1136/jnnp.54.12.1082.
[143] Toglia J, Cermak SA. Dynamic Assessment and Prediction of Learning Potential in Clients With Unilateral Neglect. Am J Occup Ther 2009;63:569–79. https://doi.org/10.5014/ajot.63.5.569.
[144] Buxbaum LJ, Coslett HB. Neglect of chimeric figures: Two halves are better than a whole. Neuropsychologia 1994;32:275–88. https://doi.org/10.1016/0028-3932(94)90131-7.
[145] Sarri M, Greenwood R, Kalra L, Papps B, Husain M, Driver J. Prism adaptation aftereffects in stroke patients with spatial neglect: Pathological effects on subjective straight ahead but not visual open-loop pointing. Neuropsychologia 2008;46:1069–80. https://doi.org/10.1016/j.neuropsychologia.2007.11.005.
[146] di Pellegrino G, Frassinetti F, Basso G. Coordinate Frames for Naming Misoriented Chimerics: A Case Study of Visuo-Spatial Neglect. Cortex 1995;31:767–77. https://doi.org/10.1016/S0010-9452(13)80027-5.
[147] Mattingley JB, Bradshaw JL, Phillips JG, Bradshaw JA. Reversed perceptual asymmetry for faces in left unilateral neglect. Brain and Cognition 1993;23:145–65. https://doi.org/10.1006/brcg.1993.1052.
[148] Massironi M, Antonucci G, Pizzamiglio L, Vitale MV, Zoccolotti P. The Wundt-Jastrow illusion in the study of spatial hemi-inattention. Neuropsychologia 1988;26:161–6.
[149] Paladini RE, Wyss P, Kaufmann BC, Urwyler P, Nef T, Cazzoli D, et al. Re-fixation and perseveration patterns in neglect patients during free visual exploration. Journal of Neuroscience 2019;49:1244–53. https://doi.org/10.1111/ejn.14309.
[150] Berger MF, Johannsen L, Karnath HO. Time Course of Eye and Head Deviation in Spatial Neglect. Neuropsychology 2008;22:697–702. https://doi.org/10.1037/a0013351.
[151] Karnath HO, Niemeier M, Dichgans J. Space exploration in neglect. Brain 1998;121 (Pt 12):2357–67. https://doi.org/10.1093/brain/121.12.2357.
[152] Kaufmann BC, Cazzoli D, Pflugshaupt T, Bohlhalter S, Vanbellingen T, Müri RM, et al. Eyetracking during free visual exploration detects neglect more reliably than paper-pencil tests. Cortex 2020;129:223–35. https://doi.org/10.1016/j.cortex.2020.04.021.
[153] Kortman B, Nicholls K. Assessing for Unilateral Spatial Neglect Using Eye-Tracking Glasses: A Feasibility Study. Occup Ther Health Care 2016;30:344–55. https://doi.org/10.1080/07380577.2016.1208858.
[154] Bourgeois A, Chica AB, Migliaccio R, de Schotten MT, Bartolomeo P. Cortical control of inhibition of return: Evidence from patients with inferior parietal damage and visual neglect. Neuropsychologia 2012;50:800–9. https://doi.org/10.1016/j.neuropsychologia.2012.01.014.
[155] Dombrowe I, Donk M, Wright H, Olivers CNL, Humphreys GW. The contribution of stimulus-driven and goal-driven mechanisms to feature-based selection in patients with spatial attention deficits. Cognitive Neuropsychology 2012;29:249–74. https://doi.org/10.1080/02643294.2012.712509.
[156] Pitteri M, Chen P, Passarini L, Albanese S, Meneghello F, Barrett AM. Conventional and functional assessment of spatial neglect: Clinical practice suggestions. Neuropsychology 2018;32:835–42. https://doi.org/10.1037/neu0000469.
[157] Bisiach E, Luzzatti C. Unilateral Neglect of Representational Space. Cortex 1978;14:129–33. https://doi.org/10.1016/S0010-9452(78)80016-1.
[158] Guariglia C, Palermo L, Piccardi L, Iaria G, Incoccia C. Neglecting the left side of a city square but not the left side of its clock: prevalence and characteristics of representational neglect. PLoS One 2013;8:e67390. https://doi.org/10.1371/journal.pone.0067390.
[159] Bartolomeo P, D'erme P, Gainotti G. The relationship between visuospatial and representational neglect. Neurology 1994;44:1710–4.
[160] Bourlon C, Pradat-Diehl P, Duret C, Azouvi P, Bartolomeo P. Seeing and imagining the "same" objects in unilateral neglect. Neuropsychologia 2008;46:2602–6. https://doi.org/10.1016/j.neuropsychologia.2008.04.002.
[161] Rode G, Revol P, Rossetti Y, Boisson D, Bartolomeo P. Looking while imagining: The influence of visual input on representational neglect. Neurology 2007;68:432–7. https://doi.org/10.1212/01.wnl.0000252936.54063.b0.
[162] Rode G, Cotton F, Revol P, Jacquin-Courtois S, Rossetti Y, Bartolomeo P. Representation and disconnection in imaginal neglect. Neuropsychologia 2010;48:2903–11. https://doi.org/10.1016/j.neuropsychologia.2010.05.032.
[163] Grossi D, Angelini R, Pecchinenda A, Pizzamiglio L. Left imaginal neglect in heminattention: experimental study with the O'clock Test. Behavioural Neurology 1993;6:155–8. https://doi.org/10.3233/BEN-1993-6307.
[164] Cocchini G, Beschin N, Jehkonen M. The Fluff Test: A simple task to assess body representation neglect. Neuropsychological Rehabilitation 2001;11:17–31. https://doi.org/10.1080/09602010042000132.
[165] Bowen A, Gardener E, Cross S, Tyrrell P, Graham J. Developing functional outcome measures for unilateral neglect: A pilot study. Neuropsychological Rehabilitation 2005;15:97–113. https://doi.org/10.1080/09602010443000010.
[166] Glocker D, Bittl P, Kerkhoff G. Construction and psychometric properties of a novel test for body representational neglect (Vest Test). Restorative Neurology and Neuroscience 2006;24:303–17.

[167] Bolognini N, Casanova D, Maravita A, Vallar G. Bisecting real and fake body parts: Effects of prism adaptation after right brain damage. Frontiers in Human Neuroscience 2012. https://doi.org/10.3389/fnhum.2012.00154.

[168] Bisiach E, Perani D, Vallar G, Berti A. Unilateral neglect: Personal and extra-personal. Neuropsychologia 1986;24:759–67. https://doi.org/10.1016/0028-3932(86)90075-8.

[169] Cunningham LJ, O'Rourke K, Finlay C, Gallagher M. A preliminary investigation into the psychometric properties of the Dublin Extrapersonal Neglect Assessment (DENA): A novel screening tool for extrapersonal neglect. Neuropsychological Rehabilitation 2017;27:349–68. https://doi.org/10.1080/09602011.2015.1084334.

[170] Borsotti M, Mosca IE, Di Lauro F, Pancani S, Bracali C, Dore T, et al. The Visual Scanning Test: a newly developed neuropsychological tool to assess and target rehabilitation of extrapersonal visual unilateral spatial neglect. Neurol Sci 2020;41:1145–52. https://doi.org/10.1007/s10072-019-04218-2.

[171] Villarreal S, Linnavuo M, Sepponen R, Vuori O, Jokinen H, Hietanen M. Dual-Task in Large Perceptual Space Reveals Subclinical Hemispatial Neglect. Journal of the International Neuropsychological Society 2020;26:993–1005. https://doi.org/10.1017/S1355617720000508.

[172] Aimola L, Schindler I, Simone AM, Venneri A. Near and far space neglect: Task sensitivity and anatomical substrates. Neuropsychologia 2012;50:1115–23. https://doi.org/10.1016/j.neuropsychologia.2012.01.022.

[173] Van Der Stoep N, Visser-Meily JMA, Kappelle LJ, De Kort PLM, Huisman KD, Eijsackers ALH, et al. Exploring near and far regions of space: Distance-specific visuospatial neglect after stroke. Journal of Clinical and Experimental Neuropsychology 2013;35:799–811. https://doi.org/10.1080/13803395.2013.824555.

[174] Berti A, Smania N, Rabuffetti M, Ferrarin M, Spinazzola L, D'Amico A, et al. Coding of far and near space during walking in neglect patients. Neuropsychology 2002;16:390–9. https://doi.org/10.1037//0894-4105.16.3.390.

[175] Ogourtsova T, Archambault PS, Lamontagne A. Post-stroke visual neglect affects goal-directed locomotion in different perceptuo-cognitive conditions and on a wide visual spectrum. Restorative Neurology and Neuroscience 2018;36:313–31. https://doi.org/10.3233/RNN-170766.

[176] Pizzamiglio L, Cappa S, Vallar G, Zoccolotti P, Bottini G, Ciurlil P, et al. Visual Neglect for Far and Near Extra-Personal Space in Humans. Cortex 1989;25:471–7. https://doi.org/10.1016/S0010-9452(89)80060-7.

[177] Whitehouse CE, Green J, Giles SM, Rahman R, Coolican J, Eskes GA. Development of the Halifax Visual Scanning Test: A new measure of visual-spatial neglect for personal, peripersonal, and extrapersonal space. Journal of the International Neuropsychological Society 2019;25:490–500. https://doi.org/10.1017/S135561771900002X.

[178] Montedoro V, Alsamour M, Dehem S, Lejeune T, Dehez B, Edwards MG. Robot Diagnosis Test for Egocentric and Allocentric Hemineglect. Archives of Clinical Neuropsychology : The Official Journal of the National Academy of Neuropsychologists 2019;34:481–94. https://doi.org/10.1093/arclin/acy062.

[179] Dawson AM, Buxbaum LJ, Rizzo AA. The virtual reality lateralized attention test: Sensitivity and validity of a new clinical tool for assessing hemispatial neglect. Virtual Rehabil., IWVR, Vancouver, BC: 2008, p. 77–82. https://doi.org/10.1109/ICVR.2008.4625140.

[180] Buxbaum LJ, Palermo MA, Mastrogiovanni D, Read MS, Rosenberg-Pitonyak E, Rizzo AA, et al. Assessment of spatial attention and neglect with a virtual wheelchair navigation task. Journal of Clinical and Experimental Neuropsychology 2008;30:650–60. https://doi.org/10.1080/13803390701625821.

[181] Fordell H, Bodin K, Bucht G, Malm J. A virtual reality test battery for assessment and screening of spatial neglect. Acta Neurologica Scandinavica 2011;123:167–74. https://doi.org/10.1111/j.1600-0404.2010.01390.x.

[182] Dvorkin AY, Bogey RA, Harvey RL, Patton JL. Mapping the neglected space: gradients of detection revealed by virtual reality. Neurorehabil Neural Repair 2012;26:120–31. https://doi.org/10.1177/1545968311410068.

[183] Peskine A, Rosso C, Box N, Galland A, Caron E, Rautureau G, et al. Virtual reality assessment for visuospatial neglect: importance of a dynamic task. Journal of Neurology, Neurosurgery and Psychiatry 2011;82:1407. https://doi.org/10.1136/jnnp.2010.217513.

[184] Kim K, Kim J, Ku J, Kim DY, Chang WH, Shin DI, et al. A Virtual Reality Assessment and Training System for Unilateral Neglect. CyberPsychology & Behavior 2004;7:742–9. https://doi.org/10.1089/cpb.2004.7.742.

[185] Kim J, Kim K, Kim DY, Chang WH, Park C-I, Ohn SH, et al. Virtual environment training system for rehabilitation of stroke patients with unilateral neglect: Crossing the virtual street. CyberPsychology & Behavior 2007;10:7–15. https://doi.org/10.1089/cpb.2006.9998.

[186] Kim DY, Ku J, Chang WH, Park TH, Lim JY, Han K, et al. Assessment of post-stroke extrapersonal neglect using a three-dimensional immersive virtual street crossing program. Acta Neurologica Scandinavica 2010;121:171–7. https://doi.org/10.1111/j.1600-0404.2009.01194.x.

[187] Navarro MD, Llorens R, Noe E, Ferri J, Alcaniz M. Validation of a low-cost virtual reality system for training street-crossing. A comparative study in healthy, neglected and non-neglected stroke individuals. Neuropsychol 2013;23:597–618. https://doi.org/10.1080/09602011.2013.806269.

[188] Ogourtsova T, Archambault P, Sangani S, Lamontagne A. Ecological Virtual Reality Evaluation of Neglect Symptoms (EVENS): Effects of Virtual Scene Complexity in the Assessment of Poststroke Unilateral Spatial Neglect. Neurorehabilitation and Neural Repair 2018;32:46–61. https://doi.org/10.1177/1545968317751677.

[189] Erez AB-H, Katz N, Ring H, Soroker N. Assessment of spatial neglect using computerised feature and conjunction visual search tasks. Neuropsychological Rehabilitation 2009;19:677–95. https://doi.org/10.1080/09602010802711160.

[190] Deouell LY, Sacher Y, Soroker N. Assessment of spatial attention after brain damage with a dynamic reaction time test. Journal of the International Neuropsychological Society 2005;11:697–707. https://doi.org/10.1017/S1355617705050824.
[191] Schendel K, Dronkers NF, Turken AU. Not Just Language: Persisting Lateralized Visuospatial Impairment after Left Hemisphere Stroke. J Int Neuropsychol Soc 8;22:695–704. https://doi.org/10.1017/S1355617716000515.
[192] List A, Brooks JL, Esterman M, Flevaris AV, Landau AN, Bowman G, et al. Visual hemispatial neglect, re-assessed. Journal of the International Neuropsychological Society 2008;14:243–56. https://doi.org/10.1017/S1355617708080284.
[193] Nyffeler T, Cazzoli D, Hess CW, Muri RM. One session of repeated parietal theta burst stimulation trains induces long-lasting improvement of visual neglect. Stroke 2009;40:2791–6. https://doi.org/10.1161/STROKEAHA.109.552323.
[194] Bublak P, Finke K, Krummenacher J, Preger R, Kyllingsbaek S, Muller HJ, et al. Usability of a theory of visual attention (TVA) for parameter-based measurement of attention II: Evidence from two patients with frontal or parietal damage. Journal of the International Neuropsychological Society 2005;11:843–54. https://doi.org/10.1017/S1355617705050988.
[195] Mazer BL, Sofer S, Korner-Bitensky N, Gelinas N. Use of the UFOV to evaluate and retrain visual attention skills in clients with stroke: A pilot study. American Journal of Occupational Therapy 2001;55:552–7. https://doi.org/10.5014/ajot.55.5.552.
[196] van Kessel ME, van Nes IJW, Geurts ACH, Brouwer WH, Fasotti L. Visuospatial asymmetry in dual-task performance after subacute stroke. J Neuropsychol 2013;7:72–90. https://doi.org/10.1111/j.1748-6653.2012.02036.x.
[197] van Kessel ME, van Nes IJW, Brouwer WH, Geurts ACH, Fasotti L. Visuospatial asymmetry and non-spatial attention in subacute stroke patients with and without neglect. Cortex 2010;46:602–12. https://doi.org/10.1016/j.cortex.2009.06.004.
[198] Bonato M, Priftis K, Marenzi R, Umiltà C, Zorzi M. Increased attentional demands impair contralesional space awareness following stroke. Neuropsychologia 2010;48:3934–40. https://doi.org/10.1016/j.neuropsychologia.2010.08.022.
[199] Bonato M. Unveiling residual, spontaneous recovery from subtle hemispatial neglect three years after stroke. Front Human Neurosci 2015;9:1–9. https://doi.org/10.3389/fnhum.2015.00413.
[200] Blini E, Romeo Z, Spironelli C, Pitteri M, Meneghello F, Bonato M, et al. Multi-tasking uncovers right spatial neglect and extinction in chronic left-hemisphere stroke patients. Neuropsychologia 2016;92:147–57. https://doi.org/10.1016/j.neuropsychologia.2016.02.028.
[201] Sacher Y, Serfaty C, Deouell L, Sapir A, Henik A, Soroker N. Role of disengagement failure and attentional gradient in unilateral spatial neglect -- a longitudinal study. Disability & Rehabilitation 2004;26:746-755 10p.
[202] D'Erme P, Robertson I, Bartolomeo P, Daniele A, Gainotti G. Early rightwards orienting of attention on simple reaction time performance in patients with left-sided neglect. Neuropsychologia 1992;30:989–1000. https://doi.org/10.1016/0028-3932(92)90050-V.
[203] Carter AR, McAvoy MP, Siegel JS, Hong X, Astafiev SV, Rengachary J, et al. Differential white matter involvement associated with distinct visuospatial deficits after right hemisphere stroke. Cortex 2017;1:81–97. https://doi.org/10.1016/j.cortex.2016.12.009.
[204] Jacquin-Courtois S, Rode G, Pavani F, O'Shea J, Giard MH, Boisson D, et al. Effect of prism adaptation on left dichotic listening deficit in neglect patients: glasses to hear better? Brain 2010;133:895–908. https://doi.org/10.1093/brain/awp327.
[205] Roberts KL, Lau JKL, Chechlacz M, Humphreys GW. Spatial and temporal attention deficits following brain injury: A neuroanatomical decomposition of the temporal order judgement task. Cognitive Neuropsychology 2012;29:300–24. https://doi.org/10.1080/02643294.2012.722548.
[206] Zimmer U, Lewald J, Karnath HO. Disturbed sound lateralization in patients with spatial neglect. Journal of Cognitive Neuroscience 2003;15:694–703. https://doi.org/10.1162/089892903322307410.
[207] Pavani F, Làdavas E, Driver J. Selective deficit of auditory localisation in patients with visuospatial neglect. Neuropsychologia 2002;40:291–301. https://doi.org/10.1016/S0028-3932(01)00091-4.
[208] Prilop L, Gutschalk A. Auditory-cortex lesions impair contralateral tone-pattern detection under informational masking. Cortex 2017;95:1–14. https://doi.org/10.1016/j.cortex.2017.07.007.
[209] Sinnett S, Juncadella M, Rafal R, Azañón E, Soto-Faraco S. A dissociation between visual and auditory hemi-inattention: Evidence from temporal order judgements. Neuropsychologia 2007;45:552–60. https://doi.org/10.1016/j.neuropsychologia.2006.03.006.
[210] Cumming TB, Blomstrand C, Bernhardt J, Linden T. The NIH stroke scale can establish cognitive function after stroke. Cerebrovasc Dis 2010;30:7–14. https://doi.org/10.1159/000313438.
[211] Maravita A, McNeil J, Malhotra P, Greenwood R, Husain M, Driver J. Prism adaptation can improve contralesional tactile perception in neglect. Neurology 2003;60:1829–31. https://doi.org/10.1212/WNL.60.11.1829.

[212] Kinsella G, Packer S, Ng K, Olver J, Stark R. Continuing issues in the assessment of neglect. Neuropsychological Rehabilitation 1995;5:239–58. https://doi.org/10.1080/09602019508401469.

[213] Anton HA, Hershler C, Lloyd P, Murray D. Visual neglect and extinction: a new test. Arch Phys Med Rehabil 1988;69:1013–6.

[214] Vossel S, Eschenbeck P, Weiss PH, Weidner R, Saliger J, Karbe H, et al. Visual extinction in relation to visuospatial neglect after right-hemispheric stroke: quantitative assessment and statistical lesion-symptom mapping. Journal of Neurology, Neurosurgery & Psychiatry 2011;82:862-868 7p. https://doi.org/10.1136/jnnp.2010.224261.

[215] Azouvi P. Functional consequences and awareness of unilateral neglect: Study of an evaluation scale. Neuropsychological Rehabilitation 1996;6:133–50. https://doi.org/10.1080/713755501.

[216] Chen P, Hreha K, Kong Y, Barrett AM. Impact of spatial neglect on stroke rehabilitation: evidence from the setting of an inpatient rehabilitation facility. Archives of Physical Medicine and Rehabilitation 2015;96:1458–66. https://doi.org/10.1016/j.apmr.2015.03.019.

[217] Chen P, Hreha K, Fortis P, Goedert K, Barrett A. Functional assessment of spatial neglect: A review of the catherine bergego scale and an introduction of the kessler foundation neglect assessment process. Topics in Stroke Rehabilitation 2012;19:423–35. https://doi.org/10.1310/tsr1905-423.

[218] Towle D, Lincoln NB. Development of a questionnaire for detecting everyday problems in stroke patients with unilateral visual neglect. Clinical Rehabilitation 1991;5:135–40. https://doi.org/10.1177/026921559100500208.

[219] Gardarsdóttir S, Kaplan S. Validity of the Arnadóttir OT-ADL Neurobehavioral Evaluation (A-ONE): performance in activities of daily living and neurobehavioral impairments of persons with left and right hemisphere damage. Am J Occup Ther 2002;56:499–508. https://doi.org/10.5014/ajot.56.5.499.

[220] Azouvi P, Olivier S, de Montety G, Samuel C, Louis-Dreyfus A, Tesio L. Behavioral assessment of unilateral neglect: study of the psychometric properties of the Catherine Bergego Scale. Arch Phys Med Rehabil 2003;84:51–7.

[221] Ten Brink AF, Verwer JH, Biesbroek J, Visser-Meily JM, Nijboer TC. Differences between left- and right-sided neglect revisited: A large cohort study across multiple domains. Journal of Clinical and Experimental Neuropsychology 2017;39:707–23. https://doi.org/10.1080/13803395.2016.1262333.

[222] Zoccolotti P, Judica A. Functional evaluation of hemineglect by means of a semistructured scale: Personal extrapersonal differentiation. Null 1991;1:33–44. https://doi.org/10.1080/09602019108401378.

[223] Zoccolotti P, Antonucci G, Judica A. Psychometric characteristics of two semi-structured scales for the functional evaluation of hemi-inattention in extrapersonal and personal space. Null 1992;2:179–91. https://doi.org/10.1080/09602019208401407.

[224] Bienkiewicz MMN, Brandi ML, Hughes C, Voitl A, Hermsdorfer J. The complexity of the relationship between neuropsychological deficits and impairment in everyday tasks after stroke. Brain and Behavior 2015;5. https://doi.org/10.1002/brb3.371.

[225] Beschin N, Robertson IH. Personal versus extrapersonal neglect: A group study of their dissociation using a reliable clinical test. Cortex 1997;33:379–84.

[226] McIntosh RD, Brodie EE, Beschin N, Robertson IH. Improving the clinical diagnosis of personal neglect: a reformulated comb and razor test. Cortex 2000;36:289–92.

[227] Levy D, Blizzard R, Halligan P, Stone S. Fluctuations in visual neglect after stroke? European Neurology 1995;35:341–3. https://doi.org/10.1159/000117157.

[228] Schwartz M, Segal M, Veramonti T, Ferraro M, Buxbaum L. The Naturalistic Action Test: a standardised assessment for everyday action impairment. Neuropsychological Rehabilitation 2002;12:311-39.

[229] Grattan ES, Woodbury ML. Do Neglect Assessments Detect Neglect Differently? Am J Occup Ther 2017;71:7103190050p1. https://doi.org/10.5014/ajot.2017.025015.

[230] Schwartz MF, Buxbaum LJ, Montgomery MW, Fitzpatrick-DeSalme E, Hart T, Ferraro M, et al. Naturalistic action production following right hemisphere stroke. Neuropsychologia 1999;37:51–66. https://doi.org/10.1016/S0028-3932%2898%2900066-9.

[231] Verlander D, Hayes A, McInnes JK, Liddle RJ, Liddle GW, Clarke GE, et al. Assessment of clients with visual spatial disorders: a pilot study. Null 2000;2:129–42. https://doi.org/10.1076/vimr.2.3.129.4422.

[232] Punt TD, Kitadono K, Hulleman J, Humphreys GW, Riddoch MJ. Modulating wheelchair navigation in patients with spatial neglect. Neuropsychological Rehabilitation 2011;21:367–82. https://doi.org/10.1080/09602011.2011.559132.

[233] Punt TD, Kitadono K, Hulleman J, Humphreys GW, Riddoch MJ. From both sides now: Crossover effects influence navigation in patients with unilateral neglect. Journal of Neurology, Neurosurgery and Psychiatry 2008;79:464–6. https://doi.org/10.1136/jnnp.2007.129205.

[234] Sjoholm H, Hagg S, Nyberg L, Rolander B, Kammerlind A-S. The Cone Evasion Walk test: Reliability and validity in acute stroke. Physiother Res Int 2019;24:e1744. https://doi.org/10.1002/pri.1744.

[235] Webster JS, Roades LA, Morrill B, Rapport LJ, Abadee PS, Sowa MV, et al. Rightward orienting bias, wheelchair maneuvering, and fall risk. Archives of Physical Medicine and Rehabilitation 1995;76:924–8. https://doi.org/10.1016/S0003-9993(95)80068-9.

[236] Webster JS, Cottam G, Gouvier WD, Blanton P, Beissel GF, Wofford J. Wheelchair obstacle course performance in right cerebral vascular accident victims. J Clin Exp Neuropsychol 1989;11:295–310.

[237] Qiang W, Sonoda S, Suzuki M, Okamoto S, Saitoh E. Reliability and validity of a wheelchair collision test for screening behavioral assessment of unilateral neglect after stroke. American Journal of Physical Medicine and Rehabilitation 2005;84:161–6. https://doi.org/10.1097/01.PHM.0000154902.79990.12.
